# The Role of Mathematical Modelling in Predicting and Controlling Infectious Disease Outbreaks in Underserved Settings: A Systematic Review and Meta‐Analysis

**DOI:** 10.1002/puh2.70116

**Published:** 2025-09-13

**Authors:** Mavhunga Khumbudzo, Evans Duah, Estelle Grobler, Kuhlula Maluleke

**Affiliations:** ^1^ School of Health Systems and Public Health Faculty of Health University of Pretoria Pretoria South Africa; ^2^ Department of Library Services University of Pretoria Pretoria South Africa

**Keywords:** disease surveillance, health equity, health system strengthening, outbreak preparedness, predictive analysis, resource‐limited settings

## Abstract

**Background and Aim:**

Mathematical modelling plays an important role in public health by enabling the prediction of disease outbreaks, assessment of transmission dynamics and evaluation of intervention strategies. Although widely applied in high‐resource settings, its use in underserved contexts remains underexplored. This review aimed to examine and synthesize current evidence on the application of mathematical modelling for predicting and controlling infectious diseases in underserved settings.

**Methods:**

A comprehensive and reproducible search was conducted using Preferred Reporting Items for Systematic Reviews and Meta‐Analyses (PRISMA) and population, intervention, comparison and outcome (PICO) frameworks across databases, including PubMed, Scopus, Medline, ScienceDirect and EBSCOhost. Keywords and Medical Subject Headings (MeSH) terms related to mathematical modelling and infectious disease control were applied. Two reviewers independently screened titles, abstracts and full texts, with a third resolving discrepancies. Thematic analysis and meta‐analysis were used for synthesis.

**Results:**

Out of 838 studies screened, 27 (3.2%) met inclusion criteria. Deterministic models were most used, followed by stochastic and agent‐based models. Diseases modelled included COVID‐19, malaria, tuberculosis (TB), Ebola, Zika, chikungunya, dengue, diphtheria, respiratory infections, visceral leishmaniasis (VL) and Mpox. Modelling predicted the impact of interventions on transmission, with pooled effect size (Ro) of 1.32 (*θ* = 1.3, *p* < 0.0001). However, challenges, such as data underreporting, gaps and inconsistencies, were common, potentially affecting model accuracy and real‐world applicability.

**Conclusion:**

Mathematical modelling has demonstrated value in supporting infectious disease control in underserved settings. However, the predominance of deterministic models limits adaptability across diverse contexts. Poor data quality further constrains reliability. Future work should focus on expanding modelling approaches, strengthening data infrastructure and addressing a broader range of diseases. These findings can guide public health policy by supporting data‐driven decision‐making, improving resource allocation and integrating modelling into outbreak preparedness and response strategies in underserved settings.

## 1 Introduction

Mathematical modelling is a powerful and increasingly indispensable tool in public health, widely used to predict, analyse and control the spread of infectious diseases [1]. By employing mathematical equations and algorithms, these models simulate the transmission dynamics of diseases within populations, allowing public health officials to understand potential outbreak scenarios and evaluate the effectiveness of various intervention strategies [1, 2]. The importance of mathematical modelling lies in its ability to provide data‐driven insights, which can inform timely and effective decision‐making [1, 2]. The World Health Organization (WHO) recognizes the critical role of modelling in enhancing global health security, particularly in developing early warning systems, guiding public health interventions and informing policy decisions during health emergencies [3].

**Research in Context**

***Evidence before this study***

Mathematical modelling has been instrumental in guiding infectious disease control, particularly in high‐income settings. However, limited attention has been given to its application and performance in underserved and resource‐constrained environments.

***Added value of this study***

This study provides a focused and novel synthesis of how infectious disease modelling has been applied in underserved settings. It summarizes the types of mathematical models used, the interventions assessed and the outcomes predicted, while also offering pooled effect estimates to support evidence‐based decision‐making in such contexts.

***Implications of all the available evidence***

The findings give the importance of tailoring modelling approaches to local conditions and strengthening modelling capacity in resource‐limited settings. This can enhance the utility of modelling in informing public health interventions and preparedness strategies where they are most needed.

Historically, mathematical modelling has played a significant role in shaping responses to major infectious disease threats, including HIV/AIDS, TB and malaria [1, 4, 5, 6]. Early pioneers such as John Graunt (1622) laid the foundations for using quantitative data to understand mortality trends [7]. In 1760, Daniel Bernoulli used modelling to define best practices for smallpox inoculation and to inform strategies [7]. In recent times, models have been central to managing global health threats such as HIV/AIDS, TB, malaria, Ebola, influenza and COVID‐19. For example, during the 2009 influenza (H1N1) pandemic, the WHO used models to help interpret outbreak data early on. Decisions based on these models informed vaccination strategies by estimating the basic reproductive number and assessing the timing and targeting of vaccinations to different groups to influence the epidemic's peak and duration [8, 9, 10]. During the West African Ebola outbreak, mathematical models estimated key parameters for outbreak control, such as the effects of case isolation, contact tracing with quarantine and sanitary funeral practices. These models also studied Ebola's spread in various settings, including communities, hospitals and traditional burial ceremonies in the Democratic Republic of the Congo (DRC) in 1995 and Uganda in 2000. The findings indicated that community transmission was a significant source of infection in Uganda, whereas traditional burial practices played a larger role in the DRC, underscoring the need for precautions to prevent transmission among patients, healthcare workers and during corpse handling [11]. For HIV prevention, modelling has evaluated the effectiveness of universal voluntary testing in reducing the HIV/AIDS epidemic as well as the effectiveness of other prevention measures, such as pre‐exposure prophylaxis (PrEP) [12, 13]. This research contributed to UNAIDS adopting the test‐and‐treat strategy aimed at the long‐term elimination of HIV [14]. Most recently, models were used to predict infection rates, hospitalizations and the impact of interventions such as lockdowns and vaccination campaigns during the COVID‐19 pandemic [6, 15].

Although the literature reflects the successful application of modelling in several high‐profile disease outbreaks, closer examination reveals several limitations, particularly in their application in underserved or resource‐limited settings. For example, models developed for high‐resource contexts during the 2009 H1N1 pandemic provided valuable insights into vaccination timing and epidemic trajectories [8, 9, 10]. However, these models were often based on comprehensive surveillance data and health infrastructure that do not exist in low‐resource settings, limiting their transferability. Similarly, during the West African Ebola outbreak, mathematical models helped estimate transmission parameters and assess the impact of control strategies like case isolation and safe burial practices [11]. Yet many of these studies, although methodologically sound, were retrospective and lacked local stakeholder engagement, raising questions about the feasibility and cultural relevance of their proposed interventions. In the context of HIV, modelling studies informed the successful adoption of test‐and‐treat strategies and the use of PrEP [12, 13, 14]. However, most of this work focused on general populations or well‐funded health systems, often overlooking the realities of implementation in fragile health environments. The COVID‐19 pandemic further illustrated the potential of modelling in predicting case surges, hospitalizations and the impact of control measures [6, 15]. Nevertheless, critical reviews of these models show issues such as overreliance on assumptions, lack of transparency and limited sensitivity analyses, especially in models applied to low‐ and middle‐income countries (LMICs) where real‐time data were either unavailable or inconsistent. Although mathematical modelling has advanced considerably in terms of sophistication and scope, its application in underserved settings remains underexplored, underreported and, in many cases, insufficiently tailored to context. Existing reviews have generally focused on theoretical developments or applications in high‐income countries, leaving a critical gap in understanding how models are applied and how effective they are in low‐resource environments where outbreaks are more frequent, consequences more severe and data, infrastructure and public health capacity are often limited.

This systematic review therefore addresses these critical gaps by synthesizing existing evidence, providing a comprehensive understanding of how mathematical models have been applied in the context of underserved settings and their effectiveness in accurately predicting outbreak dynamics and informing control strategies. Additionally, this review highlights the common challenges and barriers faced in applying mathematical modelling in underserved settings and offers recommendations for future practical applications. The findings can inform practice and policy, guiding resource allocation, intervention strategies and capacity‐building efforts to improve health outcomes in underserved populations. Furthermore, it directly contributes to achieving Sustainable Development Goal (SDG) 3 [3] by providing valuable tools for combating infectious diseases and improving health outcomes, especially in underserved populations [16, 17].

## 2 Methodology

### 2.1 Search Framework

The systematic review was conducted in adherence to the Preferred Reporting Items for Systematic Reviews and Meta‐Analyses (PRISMA) [18, 19] and the Arksey and O'Malley [20] evidence synthesis guidelines. These steps include defining the study objectives, planning the literature search, establishing eligibility criteria, screening articles for inclusion, charting the selected studies, appraising methodological quality, planning the synthesis of results and summarizing and presenting the findings. Additionally, the population, intervention, comparison and outcome (PICO) framework was employed to define the search boundaries and set the eligibility criteria (Table 1).

**TABLE 1:** Population, intervention, comparison and outcome (PICO) framework.

Population	Underserved settings globally
Intervention	The use of mathematical modelling in predicting and controlling infectious disease outbreaks
Comparison	Non‐mathematical modelling approaches to predicting and controlling infectious disease outbreaks
Outcome	Primary outcome: Accuracy of outbreak predictions, effectiveness of control measures informed by modelling Secondary outcome: Barriers to and facilitators of model implementation, best practices, and contextual factors influencing model success

### 2.2 Study Objectives

The study sought to achieve the following objectives:

1. To summarize the types of models used, diseases studied and specific outcomes measured.
2. To evaluate the effectiveness of models in predicting outbreak dynamics and informing control strategies and interventions.
3. To investigate the common challenges and barriers faced in applying mathematical modelling in underserved settings.

### 2.3 Eligibility Criteria

#### 2.3.1 Inclusion Criteria

We included:

1. Studies that focused on underserved settings, including LMICs, regions with limited healthcare infrastructure and resource‐constrained areas.
2. Studies that used mathematical models (deterministic, stochastic and/or agent‐based) to predict and control infectious disease outbreaks.
3. Studies that provide evidence on the effectiveness of the models in predicting disease spread, assessing the impact of intervention strategies and informing public health policies.
4. Peer‐reviewed articles published in scientific journals.
5. Studies published between 2014 and 2024.
6. Studies conducted in underserved settings such as LMICs.

#### 2.3.2 Exclusion Criteria

We excluded:

1. Studies conducted in high‐income countries.
2. Studies that did not utilize mathematical models.
3. Studies that focused on non‐infectious diseases.
4. Studies that did not assess the effectiveness of mathematical models in predicting or controlling infectious disease outbreaks or lack clear outcome measures.
5. Non‐peer‐reviewed articles, review articles, opinion pieces, editorials, and unpublished data.
6. Studies published outside the specified time frame or those with outdated methodologies not relevant to current modelling practices.

### 2.4 Information Sources and Search Strategy

A comprehensive and robust search was performed from major electronic databases, including PubMed, Scopus, Medline, ScienceDirect and EBSCOhost. Additionally, the bibliography and reference search were performed from the included. This snowballing technique helps identify additional relevant studies that might not have been captured in the initial database search. The search strategy involved the use of specific keywords and Medical Subject Headings (MeSH) terms related to mathematical modelling and infectious disease control. Terms, such as ‘mathematical modelling’, ‘infectious disease outbreaks’, ‘deterministic models’, ‘stochastic models’, ‘agent‐based models’, ‘public health’ and ‘underserved settings’, and names of specific diseases like ‘HIV/AIDS’, ‘TB’ and ‘malaria’ were used (Supporting Information File 1). To refine the search, Boolean operators (AND, OR, NOT) were employed to combine and exclude terms, ensuring that relevant literature is captured while minimizing irrelevant results. Search limits were applied to focus on studies published in English and within the specified time frame of 2014–2024.

### 2.5 Study Selection

**FIGURE 1:**
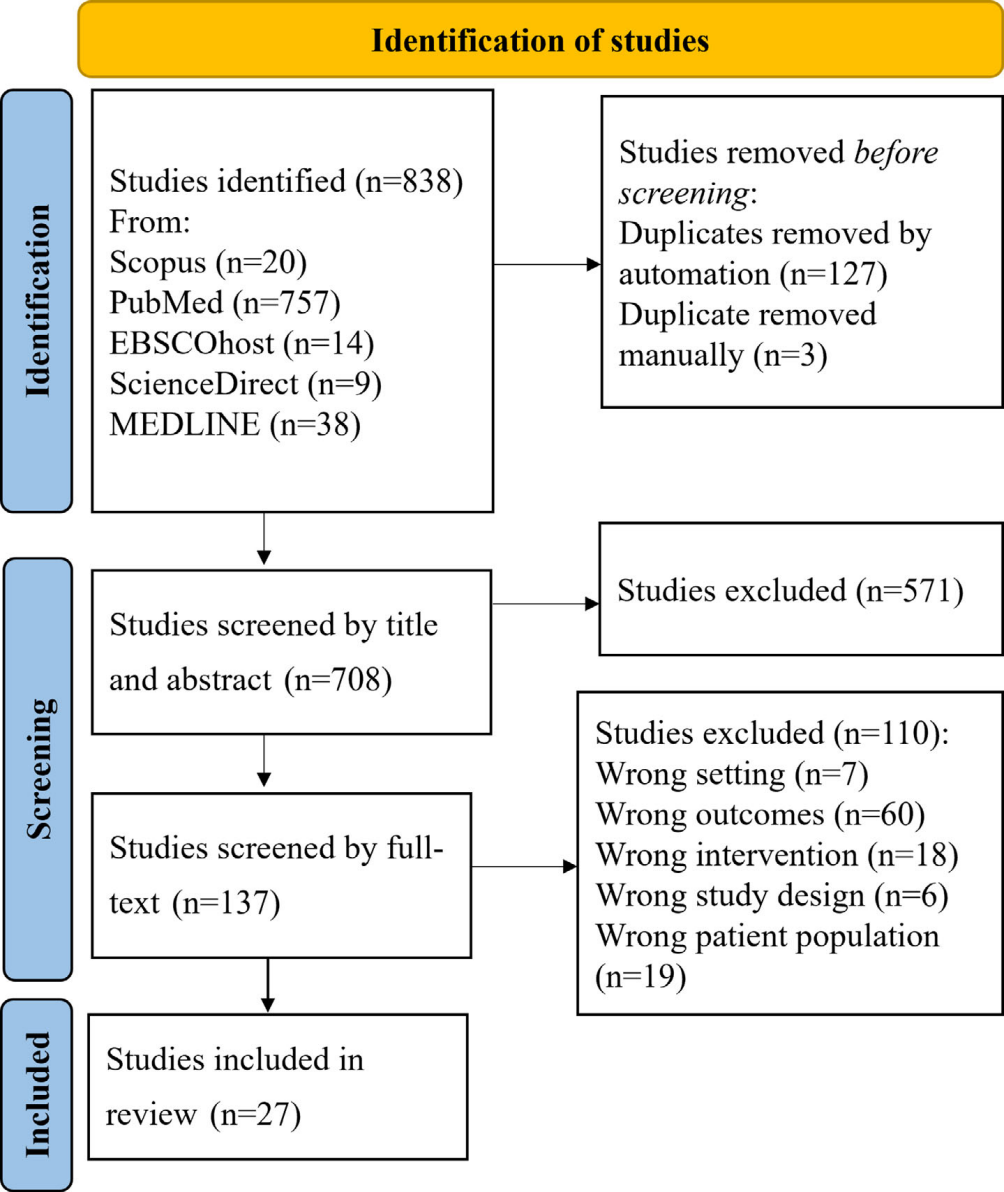
PRISMA flow chart showing literature search and number of studies included in the review.

Studies from the literature search were exported to Covidence systematic review software (Veritas Health Innovation, Melbourne, Australia) for screening [21]. Duplicates were removed both manually and automatically in Covidence. Two independent screeners conducted the initial screening of titles and abstracts, ensuring a comprehensive and unbiased selection process. Any differences in their selections were resolved through discussions between the two screeners to reach a consensus. In the second round of screening, the same independent screeners evaluated the full texts of the selected studies. Should discrepancies arise at this stage, a third screener was invited to provide additional insights and help resolve the differences. Throughout the screening process, a PRISMA flow diagram (Figure 1) was automatically populated by Covidence to document the flow of studies through the selection phases, ensuring transparency and reproducibility in the systematic review.

### 2.6 Data Extraction

Data were extracted into a predesigned form by two reviewers, who independently recorded information under the following sub‐headings and domains: author and year of publication, title of the study, aim of the study, study design, study setting, disease studied, type of mathematical model used, study findings and barriers encountered. A third reviewer was employed to resolve conflicts where there were disagreements. Corresponding authors of the included studies were contacted for additional information, data or clarification (missing or unclear information), when needed.

### 2.7 Data Charting

The extracted information was charted in a table to present relevant information on the author and year of publication, title of the study, aim of the study, study design, study setting, disease studied, type of mathematical model used, study findings and barriers encountered (Supporting Information File 2).

### 2.8 Data Analysis

#### 2.8.1 Qualitative Data Synthesis

A qualitative synthesis of the included studies was done to summarize findings using a thematic analysis approach. Thematic synthesis was conducted to identify and analyse key themes across the included studies. This involved familiarization with the data, developing themes and refining themes to capture the essence of the qualitative findings. This process was conducted manually with careful attention to details to ensure accuracy and depth of qualitative synthesis.

#### 2.8.2 Quantitative Synthesis

The quantitative data were synthesized using meta‐analysis to aggregate the findings from individual studies and provide a summary effect size. Initially, relevant quantitative data, including effect sizes (Ro), 95% confidence intervals (CIs) and standard errors, were extracted from the included study and entered into a standardized data extraction form. The data were prepared for synthesis, and data conversions were done where necessary. Data were entered in Microsoft Excel (Office version 2010) and exported to Stata 18 for meta‐analysis. We assessed heterogeneity among the included studies using the *I* ^2^ statistic and Cochran's *Q* test to determine the extent of variability in effect sizes attributable to differences between studies. We used random‐effects restricted maximum likelihood (REML) models for the meta‐analysis because no significant heterogeneity was detected among the studies. The overall effect size was estimated by computing a weighted average of the effect sizes from individual studies, with weights reflecting the precision of each study's estimate. Subgroup analyses and meta‐regression were not conducted because no heterogeneity was detected. Lastly, publication bias and outliers were assessed, and the results were presented in a funnel plot (Supporting Information File 4) and Galbraith plot (Supporting Information File 5), respectively.

## 3 Study Results

### 3.1 Screening Outcome

A total of 838 studies were identified from electronic databases: PubMed (757), MEDLINE (38), EBSCOhost (14), Scopus (20) and ScienceDirect (9) (Figure 1). The studies were exported into Covidence software, where 130 duplicates were removed. A total of 708 studies were included in title and abstract screening, where 571 were deemed irrelevant. A total of 137 studies were included for full‐text screening, and 110 were excluded from the study. The reasons for exclusion were wrong outcome (60), wrong study setting (7), wrong study design (6), wrong population (19) and wrong intervention (18). Specifically, the reasons were lack of predefined study outcome, focus on non‐mathematical modelling and lack of emphasis on underserved settings. Data were extracted from the remaining 27 articles that were included in the study.

### 3.2 Characteristics of Included Studies

**FIGURE 2:**
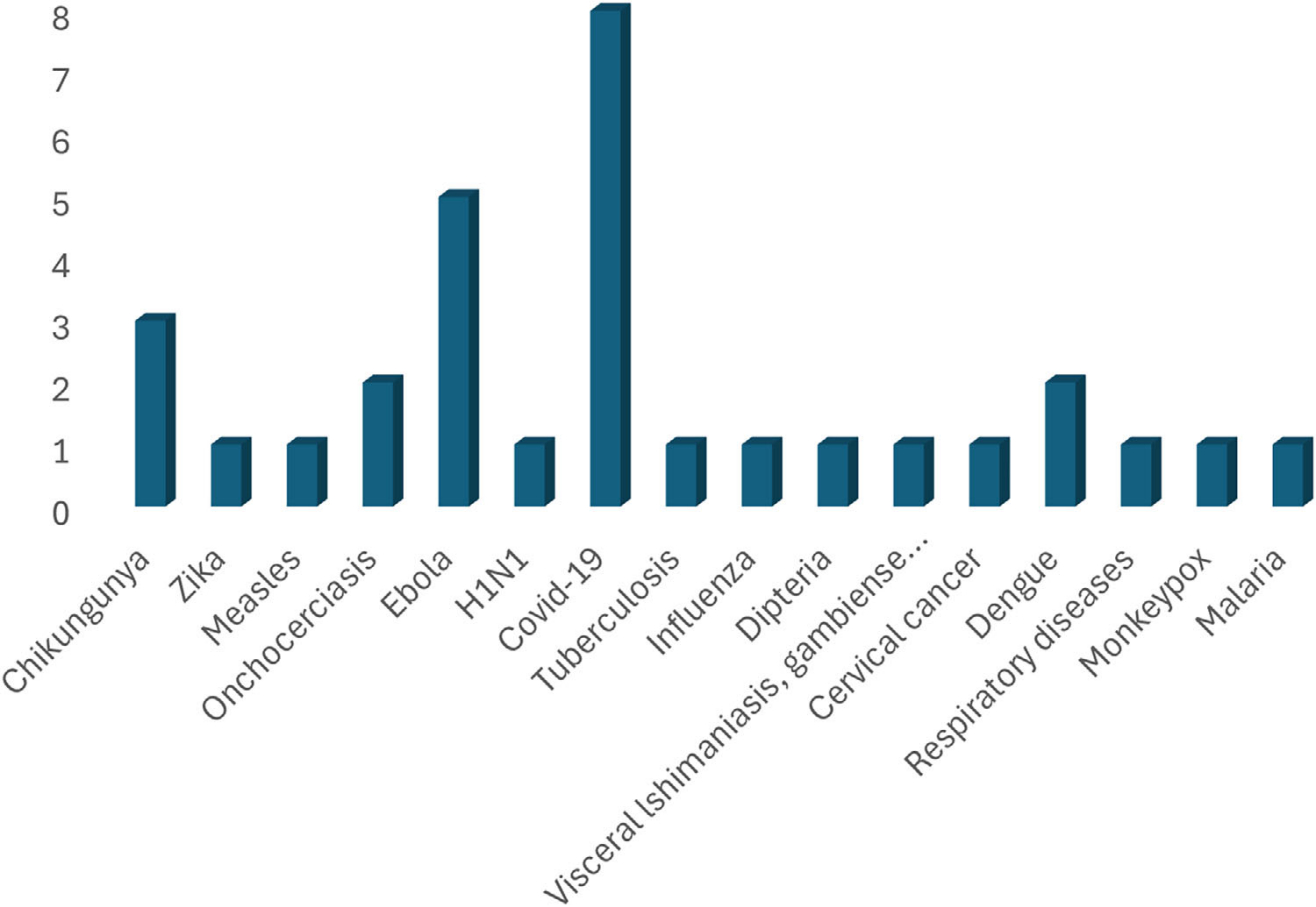
Distribution of infectious diseases modelled in studies included in the review.

**FIGURE 3:**
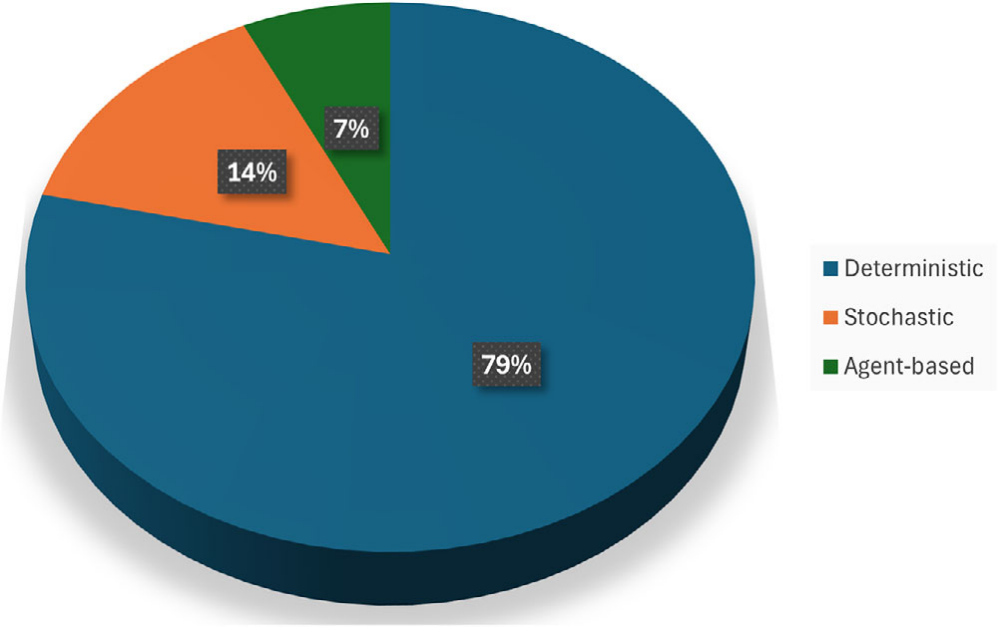
Distribution of mathematical model included in the study.

The included studies utilized mathematical modelling to examine infectious diseases such as COVID‐19, malaria, TB, Ebola, Zika, chikungunya, dengue, diphtheria, Ebola respiratory diseases, VL and Mpox. However, COVID‐19 emerged as the most studied disease using mathematical modelling, followed by Ebola (Figure 2). The commonly used models were the deterministic, stochastic and agent‐based models (Figure 3). However, the deterministic model was the most used mathematical model. The review included 27 studies conducted in various underserved settings, with the majority of the studies carried out in Africa (Supporting Information File 2). Other studies were distributed across Asia, including India, Bangladesh, China, Pakistan, Thailand, Vietnam and East Asia. In South America, studies were conducted in Brazil and Venezuela. In North America, studies were conducted in the Caribbean region and the United States. Additionally, one study was conducted in the Reunion Islands, located in the Indian Ocean.

### 3.3 Data Synthesis Outcomes

#### 3.3.1 Qualitative Data Synthesis

A qualitative synthesis of the included studies was summarized using a thematic analysis approach. Thematic synthesis was conducted to identify and analyse key themes across the included studies, reveal the gaps in knowledge and make recommendations to address the gaps. Five themes emerged, including the effectiveness of modelling, the purpose and goal of mathematical modelling, the type of model used, study findings and insights and barriers and challenges encountered.

##### 3.3.1.1 Effectiveness of Modelling

This review demonstrates that mathematical models are effective and valuable tools in helping policymakers manage outbreaks of infectious diseases, including COVID‐19, Ebola, chikungunya, dengue, Mpox, malaria and TB. For COVID‐19, models were successfully used to plan vaccination campaigns, implement quarantine measures and guide interventions to reduce the spread of the virus [22, 23, 24, 25, 26, 27, 28, 29]. In Ebola, models showed the importance of quick actions like isolating infected people and effectively performing contact tracing. Mathematical models also demonstrated the significant reduction of the spread of Ebola through the change of unsafe practices during the handling of the dead bodies of infected individuals [30, 31, 32, 33]. For Mpox, modelling showed that vaccinating more people in high‐risk groups could significantly reduce the number of infections, slow down the outbreak and make the disease less likely to spread [34]. Similarly, models for *Plasmodium vivax* malaria helped estimate how the disease spreads in certain areas, making it easier to plan control programmes [35]. For chikungunya and dengue, the study focused on how mosquitoes, such as *Aedes* species, spread the diseases and highlighted the importance of controlling mosquito populations and human behaviour to reduce cases [36]. For TB, the model pointed out ongoing challenges, particularly in endemic settings, such as South Africa, where efforts to control the disease still face significant hurdles, and developed strategies for mitigation [37].

Although mathematical modelling has been effective in helping governments and health organizations make informed decisions to reduce the spread of diseases and save lives, a key gap is that many neglected tropical diseases and diseases endemic in low‐resourced and hard‐to‐reach settings are under‐studied. More research is needed on a broader range of diseases, especially those affecting poorer or underserved populations.

##### 3.3.1.2 Purpose and Goal of Mathematical Modelling

The included studies focused on different goals related to understanding and controlling infectious diseases. Some studies looked at ways of using mathematical models to improve health interventions, such as the timing of vaccination campaigns, controlling mosquito populations for diseases like chikungunya and dengue, and managing quarantine during outbreaks [25, 38]. For instance, some studies aimed to help policymakers decide when to schedule measles immunizations, whereas another looked at how often and for how long mosquito control measures should be carried out [39, 40]. Other studies assessed the costs and effectiveness of COVID‐19 control measures, such as vaccinations and other public health actions, especially in countries with limited resources [25]. Two studies used models to predict how diseases might spread. For example, one study estimated how Ebola could have spread from affected countries to others [41], and another predicted the effects of delays in mass drug distribution for diseases like onchocerciasis [42]. Some studies focused on understanding how specific diseases, like H1N1, diphtheria and COVID‐19, spread in different environments, such as slums or areas with specific social and economic conditions [38, 43, 44].

One gap that was noticed in these studies is that many models did not consider the full complexity of real‐world situations, such as people who may spread diseases without showing symptoms or the impact of social and economic factors. More research is needed to make models more accurate, especially for resource‐constraint and disease‐endemic settings.

##### 3.3.1.3 Type of Model Used

Most of the studies included in the review used deterministic models with a few stochastic and agent‐based models. Deterministic models were commonly used because they are straightforward and provide quick results, helping researchers understand overall trends in disease outbreaks, such as how vaccination or quarantine might reduce the spread [45]. However, these models do not account for randomness or individual differences, which are important in real‐life situations. A basic model like the susceptible, infected and recovered (SIR) model is a useful starting point but fails to include important factors like vaccination coverage or the multiple sources of infection seen in diseases like Chagas [46]. Stochastic models, though used less often, are better at capturing unpredictable events and differences between individuals, making them more useful for studying small outbreaks or rare events [47]. On the other hand, agent‐based models, which were rarely used, can simulate detailed interactions between people and their surroundings, helping to understand disease patterns in specific settings like crowded urban areas or during holiday travels [48, 49].

The main gap identified in this review is the limited use of stochastic and agent‐based models that can provide deeper insights into outbreaks. Future studies should focus on using these models to improve understanding and complement deterministic approaches.

##### 3.3.1.4 Study Findings and Insights

In this review, the included studies reported on the outcomes of strategies to control infectious diseases. Some studies showed that factors like seasons, population density and age groups play a big role in the spread of diseases [23, 35]. For example, a study revealed that children and people living in crowded areas were found to contribute most to disease transmission [43]. Additionally, vaccination was highlighted as a key measure to reduce infections, especially when combined with regular booster doses, screening, and other strategies like social distancing [39, 50]. Some studies emphasized the importance of quick action, such as isolating cases during Ebola outbreaks or resuming mass drug administration (MDA) for neglected tropical diseases after disruptions [30, 33, 38, 42]. Real‐time data, like tracking people's movement through mobile phones, were also useful for predicting and managing outbreaks [51].

However, there are still gaps in knowledge. Many studies lacked detailed information on the role of people who don't show symptoms but can still spread the disease. There is also a need for better strategies to adapt interventions for low‐resource settings and more research to understand how cost and long‐term benefits can guide control efforts.

##### 3.3.1.5 Barriers and Challenges Encountered

Several studies reported data issues such as underreporting of cases, unavailability of data and inconsistent data as the major challenge [24, 26, 33, 39, 42, 46]. Models often depend on early outbreak data; however, they may be incomplete, unreliable or outdated, such as data from older Ebola outbreaks [23, 31, 37, 44]. Problems like underreporting or delays in detecting cases make it difficult to understand the true scale of outbreaks, especially in under‐resourced settings with limited surveillance systems [34]. General assumptions about how people interact may also miss local differences in behaviour or healthcare systems. Additionally, reliance on the available small number of funders due to inadequate funding institutions or groups could influence the direction of research. To improve disease modelling, more detailed, flexible and locally tailored approaches are needed to better support disease control efforts, particularly in resource‐limited settings. Another challenge highlighted was resource constraint and lack of trained personnel [40]. Some models used basic versions that do not include many real‐world details, and they might miss important parts of how diseases spread, making the results less reliable in real‐world settings [52].

#### 3.3.2 Quantitative Data Synthesis

##### 3.3.2.1 Meta‐Analysis

**FIGURE 4:**
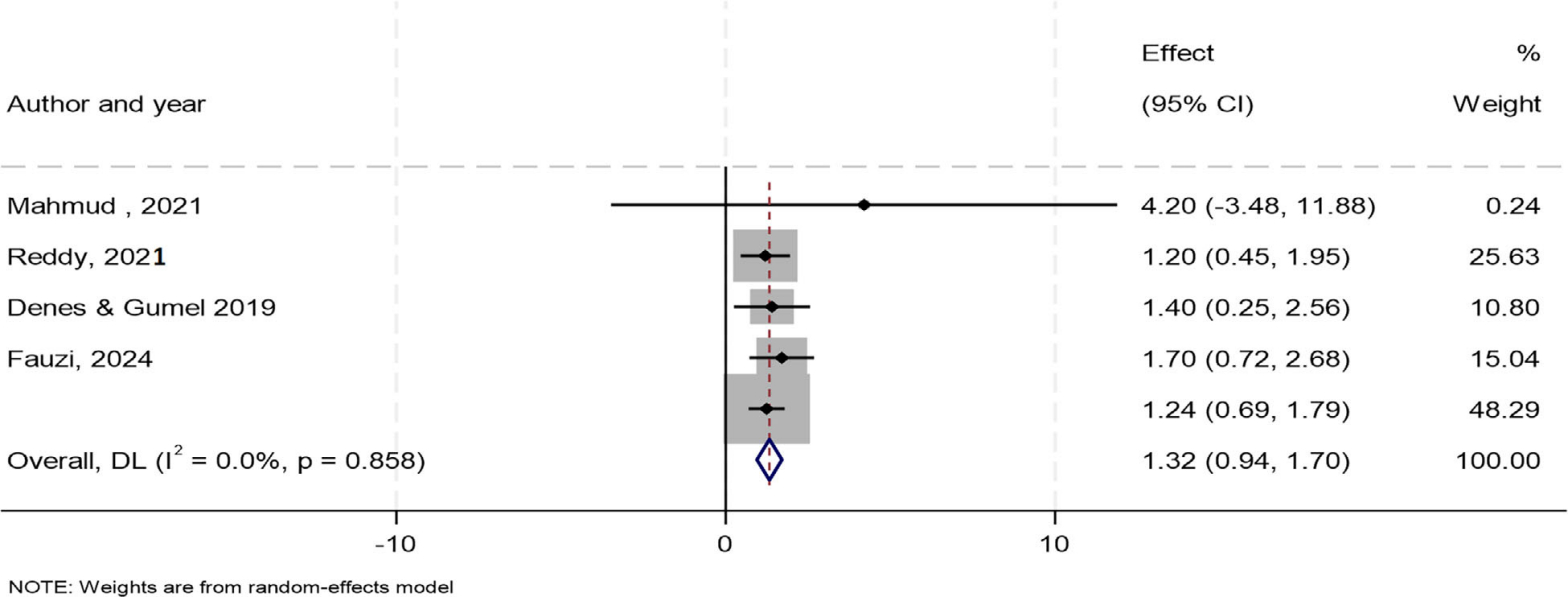
Forest plot displaying the results of individual studies and the overall pooled estimate.

**FIGURE 5:**
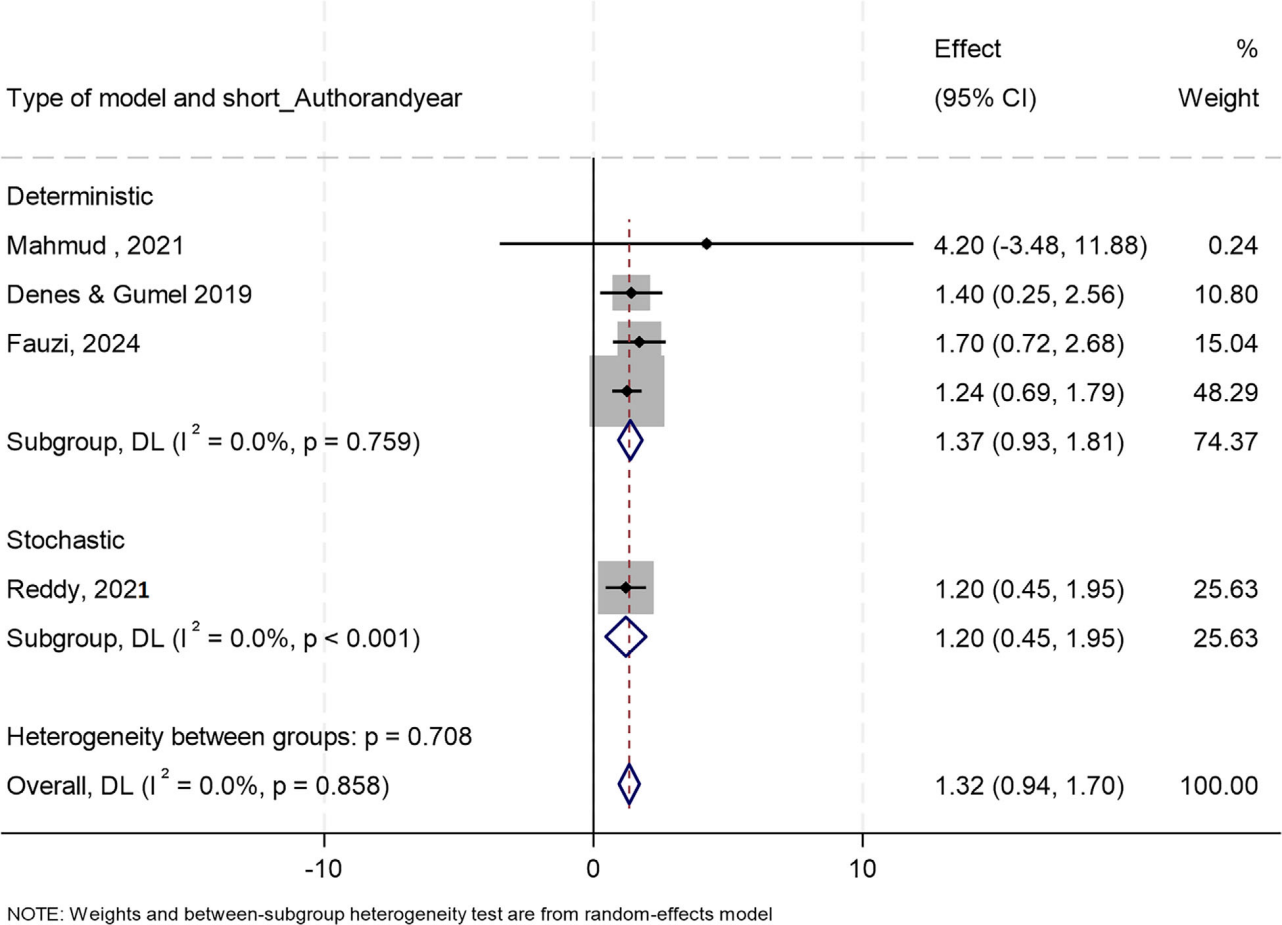
Forest plot representing subgroup analysis of heterogeneity by model type. CI, confidence interval.

Meta‐analysis was conducted to synthesize data pooled from five studies that assessed the effect of interventions for controlling disease outbreaks on the reproduction number (*R*_0_) of various diseases as predicted by mathematical models (Table 2). For example, quarantine, increasing case ascertainment, vaccination, lockdown and expanding treatment centres, among others. These studies reported effect sizes along with their 95% CIs and standard errors, indicating the level of uncertainty around each estimate. The table also outlines the type of mathematical model used and specifies the geographical setting of each study, reflecting the application of the model in an underserved setting. A random‐effects REML model was used to calculate the pooled effect size, as this approach accounts for both within‐study and between‐study variability. However, the heterogeneity analysis revealed no variability among studies as presented in Figures 4 and 5, and Supporting Information File 3.

**TABLE 2:** List of pooled studies and quantitative data for meta‐analysis.

Author [Ref.]	Reproduction or reduction number	Lower 95% CI	Upper 95% CI	Standard error	Type of model	Study setting
Mahmud [51]	4.2	3.83	4.62	3.92	Deterministic	Bangladesh
Reddy [22]	1.2	1.1	2.6	0.383	Stochastic	South Africa
Denes and Gumel [30]	1.4	0.268	2.585	0.590	Deterministic	Guinea, Liberia, Sierra Leone
Fauzi [38]	1.7	0.72	2.68	0.5	Deterministic	Indonesia
Lewnard [33]	1.24	1.18	1.29	0.279	Deterministic	Liberia

Abbreviation: CI, confidence interval.

##### 3.3.2.2 Heterogeneity Assessment

We then assessed heterogeneity among the included studies using the *I* ^2^ statistic and Cochran's *Q* test to determine the extent of variability in effect sizes attributable to differences between studies. Cochran's *Q* test yielded a *Q* value of 1.32 (*p* = 0.8585), suggesting that the variation in effect sizes was not statistically significant (Supporting Information File 3). Likewise, the *I* ^2^ value was 0.00%, indicating that there is no observable heterogeneity among the included studies (Figures 4 and 5, Supporting Information File 3). This result implies that the observed differences in effect sizes are likely due to random variation rather than systematic differences between studies. The pooled effect size was estimated to be *θ *= 1.3, and the result was statistically significant (*p* < 0.0001). This finding suggests that, on average, the interventions reduced the reproduction number across the included studies.

##### 3.3.2.3 Overall Effect Size

The forest plot demonstrates that interventions significantly reduced the reproduction number (*R*_0_) across the included studies (Figure 4). The lack of heterogeneity strengthens the confidence in the pooled effect size of 1.32. However, individual studies like Mahmud et al. [51] showed high variability, contributing minimally to the overall result. This analysis supports the effectiveness of interventions in controlling disease spread.

##### 3.3.2.4 Subgroup Analysis by Model Type

Both deterministic and stochastic models demonstrated significant reductions in the reproduction number (*R*_0_). The pooled effect size for deterministic models (1.37) is slightly higher than that for stochastic models (1.20), but the difference between the two subgroups is not statistically significant (*p* = 0.708) (Figure 5). This suggests that the effectiveness of mathematical modelling on outbreak control is robust across both deterministic and stochastic model types.

##### 3.3.2.5 Publication Bias Assessment

In this analysis, the funnel plot exhibited slight asymmetry, with one study positioned far to the right and outside the pseudo 95% confidence limits (Supporting Information File 4). This observation suggests the possibility of publication bias or the presence of methodological heterogeneity among the included studies. The asymmetry could be due to the overrepresentation of studies with significant results, particularly those with larger effect sizes. Smaller studies (with higher standard errors) showed greater dispersion, as expected due to their increased variability [53, 54].

##### 3.3.2.6 Outlier Identification by Galbraith Plot

The Galbraith plot presents the standardized effect sizes of individual studies plotted against their precision. In this plot, most studies fall within the shaded 95% CI, indicating consistency with the overall pooled effect (Supporting Information File 5). The red regression line represents the trend of the data, and the proximity of the points to this line suggests the level of agreement among studies. The black line at zero represents the no‐effect line. None of the studies deviate significantly from the shaded region, which implies minimal heterogeneity and no apparent outliers. This suggests that the meta‐analysis results are robust and reliable.

##### 3.3.2.7 Quality Appraisal

The methodological quality of included mathematical modelling studies was appraised using the checklist developed by Philips et al. for decision‐analytic models [55]. This validated checklist is made of 17 questions categorized under 4 domains: the structure of model, data, consistency and validation and uncertainty. Each question is rated NA (not applicable), 0 (not met), 1 (partially met) and 2 (fully met). The total scores are converted to percentage scores and interpreted as <60% (low quality), 60%–79% (moderate quality) and 80%–100% (high quality). Overall, 77.8% of the included studies had moderate‐to‐high quality. Specifically, 9 (33.4%) studies had high quality, 12 (44.4%) had moderate quality and 6 (22.2%) had low quality (Supporting Information File 6).

## 4 Discussions

This review synthesized current evidence on the application of mathematical modelling in predicting and managing infectious disease outbreaks, highlighting its effectiveness and potential benefits, particularly in resource‐limited settings. The deterministic model was the most employed approach, favoured for its simplicity, clarity and ease of interpretation. It effectively illustrates transitions between susceptible, infectious and recovered states within a population [45]. However, deterministic models do not account for randomness or variability, which limits their realism [45]. In contrast, stochastic models incorporate elements of chance, making them more suitable for representing systems influenced by random processes [9, 56]. On the other hand, the agent‐based models simulate behaviour and interactions of human agents to assess system‐level outcomes [48]. Despite its limitations, the deterministic model is the most utilized model in both underserved and high‐income countries. In most outbreaks, deterministic model used studied the transmission dynamics of various virulent pathogens. During the COVID‐19 pandemic, deterministic model was utilized to study the dynamics of the pandemic across the world [57, 58, 59, 60, 61, 62, 63, 64]. However, the synergistic use of two or more modelling approaches has been shown to yield deeper insights into disease transmission dynamics. For example, a study conducted in China during the COVID‐19 pandemic employed both deterministic and stochastic models to project the spread of SARS‐CoV‐2 [10]. This combined approach offered a more comprehensive understanding of the virus's behaviour and provided more robust guidance for prevention and control strategies [10].

In the current review, we found that the most extensively studied diseases were COVID‐19, Ebola, chikungunya and onchocerciasis, reflecting a focus on high‐profile infectious disease outbreaks that often receive significant global attention. Although this emphasis is understandable, it highlights a gap in research on other infectious diseases, such as malaria, TB and other neglected tropical diseases that disproportionately affect underserved communities. These diseases continue to pose persistent challenges in resource‐limited settings due to factors, such as inadequate sanitation, limited access to healthcare and broader socioeconomic inequalities [65]. Mathematical modelling efforts should expand to include these less‐studied diseases to ensure comprehensive preparedness and effective intervention planning. Doing so would enhance readiness for future outbreaks in underserved regions and help close gaps in global disease control strategies.

The meta‐analysis conducted in this study demonstrated the effectiveness of mathematical modelling in accurately predicting the impact of various public health interventions during outbreaks such as COVID‐19 and Ebola. These interventions included vaccination and mass vaccination campaigns, isolation and quarantine measures, expansion of healthcare infrastructure, MDA and the use of personal protective equipment (PPE), among others. Modelling consistently showed that these strategies contributed to a significant reduction in the basic reproduction number (*R*_0_), thereby curbing disease transmission. The findings further highlight the critical role of modelling in informing timely, evidence‐based responses, particularly in underserved settings in Bangladesh, South Africa, Guinea, Liberia, Sierra Leone and Indonesia, where the modelling was performed [22, 30, 38, 51]. In these regions, where health systems are often under‐resourced, mathematical modelling has been instrumental in supporting decision‐making, prioritizing interventions and optimizing limited resources to strengthen outbreak preparedness and control. These findings are consistent with those of a previous review, which demonstrated that public health measures such as social isolation, confinement strategies, and public education were effective in curbing the spread of respiratory infectious diseases during outbreaks and contributed to saving more lives [66]. Although the individual studies included in this review did not explicitly use modelling to arrive at these conclusions, one consistent finding is that the implementation of appropriate interventions has been effective in successfully managing and controlling infectious diseases during outbreaks.

In the current review, majority of the included studies were conducted in Africa, aligning with the WHO's emphasis on addressing health challenges in regions with high disease burdens and limited resources. Africa continues to experience recurrent infectious disease outbreaks due to factors, such as weak healthcare systems, inadequate sanitation and limited access to vaccination [67, 68]. In response, the WHO prioritizes capacity building in these settings by promoting disease surveillance, outbreak preparedness and the use of mathematical modelling to support evidence‐based decision‐making. The concentration of research in Africa suggests that the scientific community is actively responding to global health priorities, aiming to enhance the capacity to predict, respond to and control outbreaks in resource‐limited settings [16, 17]. However, a major challenge in the African context is the heavy reliance on outdated outbreak data, alongside persistent issues such as underreporting of cases, limited data availability, and inconsistencies in data quality [24, 26, 33, 39, 42, 46]. These factors significantly constrain the accuracy and effectiveness of mathematical modelling efforts across the continent.

### 4.1 Strength and Limitations

The strength of this review lies in its systematic, comprehensive and reproducible search strategy, which ensured broad coverage of relevant literature. A robust quality appraisal was conducted, and the included studies showed minimal heterogeneity and limited risk of bias, enhancing the credibility of the findings. Additionally, the inclusion of a substantial number of studies that met the inclusion criteria strengthens the overall evidence base and supports the generalizability of the narrative synthesis. A key limitation is the inclusion of six studies that were rated as low quality due to the absence of clear information on essential methodological elements such as data sources, procedures for data identification, assessment of data quality and relevance, justification for data treatment (e.g., extrapolation or transformation), internal model validation (e.g., debugging), external validation and the handling of uncertainty (e.g., sensitivity analysis, structural or methodological uncertainty, discounting and time horizons). However, these were only 6 out of 27 included studies, representing 22.2% of the included studies. Despite their poor quality, they were retained because they addressed the core objectives of the review. Excluding these studies would have risked omitting valuable context‐specific insights from resource‐limited settings, where methodological reporting is often constrained, and the overall body of evidence remains limited. To mitigate the potential bias associated with their lower quality, we applied a structured and transparent quality appraisal process and clearly documented the limitations of these studies. Additionally, findings from these studies were interpreted with caution and were not overemphasized in our conclusions. All claims have been cross‐validated against higher‐quality studies in the discussion to ensure that any policy‐relevant or practice‐informed insights were grounded in consistency. Additionally, only five studies were eligible for inclusion in the meta‐analysis, as they provided pooled data on reproduction numbers during disease outbreaks. This limited quantitative analysis reduces the ability to draw strong statistical conclusions about intervention effectiveness across diverse settings. The limited number of quantitative studies may limit the statistical power and preclude broad generalizability of the meta‐analysis. However, this reflects the scarcity of quantitative modelling research conducted in underserved settings, a critical gap this review aims to highlight. Although the pooled estimates provide valuable insights, they should be interpreted with caution and within the context of the specific populations and diseases studied. Furthermore, the dominance of deterministic models and the underrepresentation of diseases beyond high‐profile outbreaks such as COVID‐19 and Ebola may limit the applicability of findings to a broader range of infectious diseases, particularly those endemic in underserved populations.

### 4.2 Conclusions

This review provides evidence of how mathematical modelling has been applied to support the prediction and control of infectious disease outbreaks in underserved populations. Although several studies demonstrated practical utility, the effectiveness of modelling approaches varied across contexts. Notably, one consistent finding was the potential of modelling to predict the impact of interventions on reducing reproduction numbers (*R*_0_), as demonstrated by the meta‐analysis, which yielded a pooled effect size of 1.32 (*θ* = 1.32, *p* < 0.0001). Most of the included studies employed deterministic models, valued for their simplicity and interpretability, but these models offered limited variability and may constrain broader applicability. The reliance on a single modelling approach in many cases highlights the need for methodological diversity to enhance contextual relevance. Importantly, the review highlights persistent challenges in data availability and quality, which can significantly affect the reliability of modelling outcomes. This work contributes to ongoing efforts aligned with SDG 3 by promoting evidence‐informed approaches to disease prevention in low‐resource settings. This also supports SDG 10 by highlighting the need to reduce health inequities through improved access to data‐driven public health tools.

### 4.3 Recommendation for Research

To address the limitations outlined, future research should prioritize the exploration of diverse modelling techniques, strengthen data collection and reporting systems and broaden the scope to include a wider range of diseases affecting underserved populations. Additionally, there is a need for more high‐quality, quantitative studies that evaluate model performance using real‐world data. To enhance the quality and practical relevance of future modelling studies in underserved settings, we recommend the adoption of standardized modelling and reporting frameworks.

### 4.4 Recommendations for Practice

#### 4.4.1 Strengthening Data Collection and Management Systems

An effective data collection and management system is essential for generating accurate and reliable information to support public health decision‐making. This can be achieved by providing targeted training to healthcare workers, equipping them with the necessary skills and knowledge to manage and utilize data effectively. Additionally, strengthening local infrastructure, such as implementing robust health information systems and disease surveillance networks, can enhance the timeliness and quality of data collection, particularly in resource‐limited settings. Public health practitioners and policymakers in underserved areas should also prioritize the integration of context‐specific mathematical modelling approaches to enhance outbreak preparedness and guide response strategies.

#### 4.4.2 Leveraging Technology

Mobile phones, mobile applications and electronic health records offer practical tools for gathering, storing and transmitting data efficiently, particularly in remote and underserved areas. These technologies enable real‐time data collection, which can significantly enhance response times. Moreover, they facilitate the tracking of disease trends and support the early detection of outbreaks, thereby strengthening overall surveillance and public health response systems. These provide adequate and quality data for models.

## Author Contributions

**Mavhunga Khumbudzo**: conceptualization, investigation, methodology, data curation, formal analysis, visualization, project administration, resources, writing – original draft. **Evans Duah**: methodology, data curation, investigation, validation, writing – review and editing. **Estelle Grobler**: methodology, software, data curation, investigation, validation. **Kuhlula Maluleke**: supervision, conceptualization, methodology, investigation, validation, writing – review and editing.

## Disclosure

This systematic review protocol was registered in PROSPERO (registration number: CRD420251044261).

## Ethics Statement

The authors have nothing to report.

## Conflicts of Interest

The authors declare no conflicts of interest.

## Supporting information




**Supplementary file 1**. Search strategy

**Supplementary file 2**. Characteristics and findings of the studies included in the review

**Supplementary file 3**. Summary of meta‐analysis results

**Supplementary file 4**: Funnel plot representing publication bias assessment

**Supplementary file 5**. Galbraith plot to identify outliers

**Supplementary file 6**. Quality appraisal

## Data Availability

All data used in this study are provided as Supporting Information.

## References

[1] M. Kretzschmar , “Disease Modeling for Public Health: Added Value, Challenges, and Institutional Constraints,” Journal of Public Health Policy 41, no. 1 (2020): 39–51. 31780754 10.1057/s41271-019-00206-0 PMC7041603

[2] S. Greenhalgh and C. Rozins , “A Generalized Differential Equation Compartmental Model of Infectious Disease Transmission,” Infectious Disease Modelling 6 (2021): 1073–1091. 34585030 10.1016/j.idm.2021.08.007 PMC8449186

[3] M. Egger , L. Johnson , C. Althaus , et al., “Developing WHO Guidelines: Time to Formally Include Evidence From Mathematical Modelling Studies,” F1000Research 6 (2017): 1584. 29552335 10.12688/f1000research.12367.1 PMC5829466

[4] A. S. Bhadauria , H. N. Dhungana , V. Verma , S. Woodcock , and T. Rai , “Studying the Efficacy of Isolation as a Control Strategy and Elimination of Tuberculosis in India: A Mathematical Model,” Infectious Disease Modelling 8, no. 2 (2023): 458–470. 37234098 10.1016/j.idm.2023.03.005 PMC10206434

[5] O. C. Collins and K. J. Duffy , “A Mathematical Model for the Dynamics and Control of Malaria in Nigeria,” Infectious Disease Modelling 7, no. 4 (2022): 728–741. 36407847 10.1016/j.idm.2022.10.005 PMC9661649

[6] W. M. Sweileh , “Global Research Activity on Mathematical Modeling of Transmission and Control of 23 Selected Infectious Disease Outbreak,” Global Health 18, no. 1 (2022): 4. 35062966 10.1186/s12992-022-00803-x PMC8778503

[7] F. Brauer , “Mathematical Epidemiology: Past, Present, and Future,” Infectious Disease Modelling 2, no. 2 (2017): 113–127. 29928732 10.1016/j.idm.2017.02.001 PMC6001967

[8] N. Wang , Y. Fu , H. Zhang , and H. Shi , “An Evaluation of Mathematical Models for the Outbreak of COVID‐19,” Precision Clinical Medicine 3, no. 2 (2020): 85–93. 35960670 10.1093/pcmedi/pbaa016 PMC7376265

[9] E. Cuevas , “An Agent‐Based Model to Evaluate the COVID‐19 Transmission Risks in Facilities,” Computers in Biology and Medicine 121 (2020): 103827. 32568667 10.1016/j.compbiomed.2020.103827 PMC7237380

[10] D. Olabode , J. Culp , A. Fisher , A. Tower , D. Hull‐Nye , and X. Wang , “Deterministic and Stochastic Models for the Epidemic Dynamics of COVID‐19 in Wuhan, China,” Mathematical Biosciences and Engineering 18, no. 1 (2021): 950–967. 33525127 10.3934/mbe.2021050

[11] J. Legrand , R. F. Grais , P.‐Y. Boelle , A.‐J. Valleron , and A. Flahault , “Understanding the Dynamics of Ebola Epidemics,” Epidemiology & Infection 135, no. 4 (2007): 610–621. 16999875 10.1017/S0950268806007217 PMC2870608

[12] C. W. Dieffenbach and A. S. Fauci , “Universal Voluntary Testing and Treatment for Prevention of HIV Transmission,” JAMA 301, no. 22 (2009): 2380–2382. 19509386 10.1001/jama.2009.828

[13] S. B. Kim , M. Yoon , N. S. Ku , et al., “Mathematical Modeling of HIV Prevention Measures Including Pre‐Exposure Prophylaxis on HIV Incidence in South Korea,” PLoS ONE 9, no. 3 (2014): e90080. 24662776 10.1371/journal.pone.0090080 PMC3963840

[14] D. S. Boakye and S. Adjorlolo , “Achieving the UNAIDS 95‐95‐95 Treatment Target by 2025 in Ghana: A Myth or a Reality?,” Global Health Action 16, no. 1 (2023): 2271708. 37921654 10.1080/16549716.2023.2271708 PMC10627043

[15] A. Adiga , D. Dubhashi , B. Lewis , M. Marathe , S. Venkatramanan , and A. Vullikanti , “Mathematical Models for Covid‐19 Pandemic: A Comparative Analysis,” Journal of the Indian Institute of Science 100, no. 4 (2020): 793–807. 33144763 10.1007/s41745-020-00200-6 PMC7596173

[16] World Health Organization , Health in 2015: From MDGs, Millennium Development Goals to SDGs, Sustainable Development Goals (World Health Organization, 2015).

[17] M. Egger , L. Johnson , C. Althaus , et al., “Developing WHO Guidelines: Time to Formally Include Evidence From Mathematical Modelling Studies,” F1000Research 6 (2017): 1584. 29552335 10.12688/f1000research.12367.1 PMC5829466

[18] M. L. Rethlefsen and M. J. Page , “PRISMA 2020 and PRISMA‐S: Common Questions on Tracking Records and the Flow Diagram,” Journal of the Medical Library Association: JMLA 110, no. 2 (2022): 253–257. 35440907 10.5195/jmla.2022.1449 PMC9014944

[19] C. Sohrabi , T. Franchi , G. Mathew , et al., PRISMA 2020 Statement: What's New and the Importance of Reporting Guidelines (Elsevier, 2021). 10.1016/j.ijsu.2021.105918 33789825

[20] H. Arksey and L. O'malley , “Scoping Studies: Towards a Methodological Framework,” International Journal of Social Research Methodology 8, no. 1 (2005): 19–32.

[21] V. Innovation , Covidence—Better Systematic Review Management (Veritas Health Innovation, 2018).

[22] K. P. Reddy , F. M. Shebl , J. H. A. Foote , et al., “Cost‐Effectiveness of Public Health Strategies for COVID‐19 Epidemic Control in South Africa: A Microsimulation Modelling Study,” Lancet Global Health 9, no. 2 (2021): e120–e129. 33188729 10.1016/S2214-109X(20)30452-6 PMC7834260

[23] S. Nagpal , R. Kumar , R. F. Noronha , et al., “Seasonal Variations in Social Contact Patterns in a Rural Population in North India: Implications for Pandemic Control,” PLoS ONE 19, no. 2 (2024): e0296483. 38386667 10.1371/journal.pone.0296483 PMC10883557

[24] M. A. Kuddus , A. K. Paul , and T. Theparod , “Cost‐Effectiveness Analysis of COVID‐19 Intervention Policies Using a Mathematical Model: An Optimal Control Approach,” Scientific Reports 14, no. 1 (2024): 494. 38177230 10.1038/s41598-023-50799-6 PMC10766655

[25] C. A. B. Pearson , F. Bozzani , S. R. Procter , et al., “COVID‐19 Vaccination in Sindh Province, Pakistan: A Modelling Study of Health Impact and Cost‐Effectiveness,” PLoS Medicine 18, no. 10 (2021): e1003815. 34606520 10.1371/journal.pmed.1003815 PMC8523052

[26] Y. Baik , L. Cilloni , E. Kendall , D. Dowdy , and N. Arinaminpathy , “Symptom‐Based vs Asymptomatic Testing for Controlling SARS‐CoV‐2 Transmission in Low‐ and Middle‐Income Countries: A Modelling Analysis,” Epidemics 41 (2022): 100631. 36174427 10.1016/j.epidem.2022.100631 PMC9511882

[27] K. van Zandvoort , C. I. Jarvis , C. A. B. Pearson , et al., “Response Strategies for COVID‐19 Epidemics in African Settings: A Mathematical Modelling Study,” BMC Medicine [Electronic Resource] 18, no. 1 (2020): 324. 33050951 10.1186/s12916-020-01789-2 PMC7553800

[28] M. Ladib , A. Ouhinou , and A. A. Yakubu , “Mathematical Modeling of Contact Tracing and Stability Analysis to Inform Its Impact on Disease Outbreaks; an Application to COVID‐19,” Infectious Disease Modelling 9, no. 2 (2024): 329–353. 38371875 10.1016/j.idm.2024.01.010 PMC10867662

[29] Y. Liu , S. R. Procter , C. A. B. Pearson , et al., “Assessing the Impacts of COVID‐19 Vaccination Programme's Timing and Speed on Health Benefits, Cost‐Effectiveness, and Relative Affordability in 27 African Countries,” BMC Medicine [Electronic Resource] 21, no. 1 (2023): 85. 36882868 10.1186/s12916-023-02784-z PMC9991879

[30] A. Denes and A. B. Gumel , “Modeling the Impact of Quarantine During an Outbreak of Ebola Virus Disease,” Infectious Disease Modelling 4 (2019): 12–27. 30828672 10.1016/j.idm.2019.01.003 PMC6382747

[31] B. Levy , C. Edholm , O. Gaoue , et al., “Modeling the Role of Public Health Education in Ebola Virus Disease Outbreaks in Sudan,” Infectious Disease Modelling 2, no. 3 (2017): 323–340. 29928745 10.1016/j.idm.2017.06.004 PMC6001965

[32] S. D. Djiomba Njankou and F. Nyabadza , “Modelling the Potential Influence of Human Migration and Two Strains on Ebola Virus Disease Dynamics,” Infectious Disease Modelling 7, no. 4 (2022): 645–659. 36313151 10.1016/j.idm.2022.10.002 PMC9583178

[33] J. A. Lewnard , M. L. Ndeffo Mbah , J. A. Alfaro‐Murillo , et al., “Dynamics and Control of Ebola Virus Transmission in Montserrado, Liberia: A Mathematical Modelling Analysis,” Lancet Infectious Diseases 14, no. 12 (2014): 1189–1195. 25455986 10.1016/S1473-3099(14)70995-8 PMC4316822

[34] H. K. Das , “Exploring the Dynamics of Monkeypox Transmission With Data‐Driven Methods and a Deterministic Model,” Frontiers in Epidemiology 4 (2024): 1334964. 38840980 10.3389/fepid.2024.1334964 PMC11150605

[35] B. Shi , S. Lin , Q. Tan , et al., “Inference and Prediction of Malaria Transmission Dynamics Using Time Series Data,” Infectious Diseases of Poverty 9, no. 1 (2020): 95. 32678025 10.1186/s40249-020-00696-1 PMC7367373

[36] C. A. Manore , K. S. Hickmann , S. Xu , H. J. Wearing , and J. M. Hyman , “Comparing Dengue and Chikungunya Emergence and Endemic Transmission in *A. aegypti* and *A. albopictus* ,” Journal of Theoretical Biology 356 (2014): 174–191. 24801860 10.1016/j.jtbi.2014.04.033 PMC4109365

[37] A. Azeez , D. Obaromi , A. Odeyemi , J. Ndege , and R. Muntabayi , “Seasonality and Trend Forecasting of Tuberculosis Prevalence Data in Eastern Cape, South Africa, Using a Hybrid Model,” International Journal of Environmental Research and Public Health 13, no. 8 (2016): 757. 27472353 10.3390/ijerph13080757 PMC4997443

[38] I. S. Fauzi , N. Nuraini , A. M. Sari , et al., “Assessing the Impact of Booster Vaccination on Diphtheria Transmission: Mathematical Modeling and Risk Zone Mapping,” Infectious Disease Modelling 9, no. 1 (2024): 245–262. 38312350 10.1016/j.idm.2024.01.004 PMC10837633

[39] S. Verguet , M. Johri , S. K. Morris , C. L. Gauvreau , P. Jha , and M. Jit , “Controlling Measles Using Supplemental Immunization Activities: A Mathematical Model to Inform Optimal Policy,” Vaccine 33, no. 10 (2015): 1291–1296. 25541214 10.1016/j.vaccine.2014.11.050 PMC4336184

[40] N. J. Lemanski , S. R. Schwab , D. M. Fonseca , and N. H. Fefferman , “Coordination Among Neighbors Improves the Efficacy of Zika Control Despite Economic Costs,” PLOS Neglected Tropical Diseases 14, no. 6 (2020): e0007870. 32569323 10.1371/journal.pntd.0007870 PMC7332071

[41] J. I. D. Hamley , D. J. Blok , M. Walker , et al., “What Does the COVID‐19 Pandemic Mean for the Next Decade of Onchocerciasis Control and Elimination?,” Transactions of the Royal Society of Tropical Medicine and Hygiene 115, no. 3 (2021): 269–280. 33515042 10.1093/trstmh/traa193 PMC7928565

[42] I. Routledge , M. Walker , R. A. Cheke , et al., “Modelling the Impact of Larviciding on the Population Dynamics and Biting Rates of *Simulium damnosum* (s.l.): Implications for Vector Control as a Complementary Strategy for Onchocerciasis Elimination in Africa,” Parasites & Vectors 11, no. 1 (2018): 316. 29843770 10.1186/s13071-018-2864-y PMC5972405

[43] A. Adiga , S. Chu , S. Eubank , et al., “Disparities in Spread and Control of Influenza in Slums of Delhi: Findings From an Agent‐Based Modelling Study,” BMJ Open 8, no. 1 (2018): e017353. 10.1136/bmjopen-2017-017353 PMC5780711 29358419

[44] P. Pongsumpun and I. M. Tang , “Dynamics of a New Strain of the H1N1 Influenza A Virus Incorporating the Effects of Repetitive Contacts,” Computational and Mathematical Methods in Medicine 2014 (2014): 487974. 24744816 10.1155/2014/487974 PMC3973015

[45] M. Roberts , V. Andreasen , A. Lloyd , and L. Pellis , “Nine Challenges for Deterministic Epidemic Models,” Epidemics 10 (2015): 49–53. 25843383 10.1016/j.epidem.2014.09.006 PMC4996659

[46] K. S. Rock , L. A. C. Chapman , A. P. Dobson , E. R. Adams , and T. D. Hollingsworth , “The Hidden Hand of Asymptomatic Infection Hinders Control of Neglected Tropical Diseases: A Modeling Analysis,” Clinical Infectious Diseases 78, no. S2 (2024): S175–S182. 38662705 10.1093/cid/ciae096 PMC11045017

[47] C. da Anderson Luiz Pena , P. Marcelo Amanajas , R. Rafael Lima , and A. da Sheylla Susan Moreira Silva De , “Mathematical Modeling of the Infectious Diseases: Key Concepts and Applications,” Journal of Infectious Diseases and Epidemiology 7, no. 5 (2021): 209.

[48] Y. Li , M. A. Lawley , D. S. Siscovick , D. Zhang , and J. A. Pagan , “Agent‐Based Modeling of Chronic Diseases: A Narrative Review and Future Research Directions,” Preventing Chronic Disease [Electronic Resource] 13 (2016): E69. 27236380 10.5888/pcd13.150561 PMC4885681

[49] E. Hunter , B. Mac Namee , and J. Kelleher , “An Open‐Data‐Driven Agent‐Based Model to Simulate Infectious Disease Outbreaks,” PLoS ONE 13, no. 12 (2018): e0208775. 30566424 10.1371/journal.pone.0208775 PMC6300276

[50] M. Drolet , J. F. Laprise , D. Martin , et al., “Optimal Human Papillomavirus Vaccination Strategies to Prevent Cervical Cancer in Low‐Income and Middle‐Income Countries in the Context of Limited Resources: A Mathematical Modelling Analysis,” Lancet Infectious Diseases 21, no. 11 (2021): 1598–1610. 34245682 10.1016/S1473-3099(20)30860-4 PMC8554391

[51] A. S. Mahmud , M. I. Kabir , K. Engø‐Monsen , et al., “Megacities as Drivers of National Outbreaks: The 2017 Chikungunya Outbreak in Dhaka, Bangladesh,” PLOS Neglected Tropical Diseases 15, no. 2 (2021): e0009106. 33529229 10.1371/journal.pntd.0009106 PMC7880496

[52] T. Fung , J. Goh , and R. A. Chisholm , “Long‐Term Effects of Non‐Pharmaceutical Interventions on Total Disease Burden in Parsimonious Epidemiological Models,” Journal of Theoretical Biology 587 (2024): 111817. 38599566 10.1016/j.jtbi.2024.111817

[53] J. A. Naslund , K. L. Whiteman , G. J. McHugo , K. A. Aschbrenner , L. A. Marsch , and S. J. Bartels , “Lifestyle Interventions for Weight Loss Among Overweight and Obese Adults With Serious Mental Illness: A Systematic Review and Meta‐Analysis,” General Hospital Psychiatry 47 (2017): 83–102. 28807143 10.1016/j.genhosppsych.2017.04.003 PMC5575752

[54] G. Mohammed Mustafa and C. Chandana , “A Meta‐Analysis on Misuse of Prescription/OTC Drugs: How Pharmacist Can Prevent and Manage Drug Abuse,” Indian Journal of Pharmaceutical Education and Research 57, no. 1s (2023): S167–S173.

[55] Z. Philips , L. Ginnelly , M. Sculpher , et al., “Review of Guidelines for Good Practice in Decision‐Analytic Modelling in Health Technology Assessment,” Health Technology Assessment (Winchester, England) 8, no. 36 (2004): iii–iv, ix. 10.3310/hta8360 15361314

[56] J. B. Xavier , J. M. Monk , S. Poudel , et al., “Mathematical Models to Study the Biology of Pathogens and the Infectious Diseases They Cause,” Iscience 25, no. 4 (2022): 104079. 35359802 10.1016/j.isci.2022.104079 PMC8961237

[57] R. Prasad , S. K. Sagar , S. Parveen , and R. Dohare , “Mathematical Modeling in Perspective of Vector‐Borne Viral Infections: A Review,” Beni‐Suef University Journal of Basic and Applied Sciences 11, no. 1 (2022): 102. 36000145 10.1186/s43088-022-00282-4 PMC9388993

[58] L. Pinky and H. M. Dobrovolny , “Epidemiological Consequences of Viral Interference: A Mathematical Modeling Study of Two Interacting Viruses,” Frontiers in Microbiology 13 (2022): 830423. 35369460 10.3389/fmicb.2022.830423 PMC8966706

[59] P. Jimenez‐Rodriguez , G. A. Munoz‐Fernandez , J. C. Rodrigo‐Chocano , J. B. Seoane‐Sepulveda , and A. Weber , “A Population Structure‐Sensitive Mathematical Model Assessing the Effects of Vaccination During the Third Surge of COVID‐19 in Italy,” Journal of Mathematical Analysis and Applications 514, no. 2 (2022): 125975. 35001969 10.1016/j.jmaa.2021.125975 PMC8717707

[60] T. T. Marinov and R. S. Marinova , “Inverse Problem for Adaptive SIR Model: Application to COVID‐19 in Latin America,” Infectious Disease Modelling 7, no. 1 (2022): 134–148. 34934870 10.1016/j.idm.2021.12.001 PMC8674112

[61] H. De‐Leon and D. Aran , “MAM: Flexible Monte‐Carlo Agent Based Model for Modelling COVID‐19 Spread,” Journal of Biomedical Informatics 141 (2023): 104364. 37061013 10.1016/j.jbi.2023.104364 PMC10098313

[62] J. Wang , “Mathematical Models for Cholera Dynamics—A Review,” Microorganisms 10, no. 12 (2022): 2358. 36557611 10.3390/microorganisms10122358 PMC9783556

[63] A. Wiratsudakul , P. Suparit , and C. Modchang , “Dynamics of Zika Virus Outbreaks: An Overview of Mathematical Modeling Approaches,” PeerJ 6 (2018): e4526. 29593941 10.7717/peerj.4526 PMC5866925

[64] M. R. K. Ariffin , K. Gopal , I. Krishnarajah , et al., “Mathematical Epidemiologic and Simulation Modelling of First Wave COVID‐19 in Malaysia,” Scientific Reports 11, no. 1 (2021): 20739. 34671103 10.1038/s41598-021-99541-0 PMC8528817

[65] M. De Rycker , B. Baragaña , S. L. Duce , and I. H. Gilbert , “Challenges and Recent Progress in Drug Discovery for Tropical Diseases,” Nature 559, no. 7715 (2018): 498–506. 30046073 10.1038/s41586-018-0327-4 PMC6129172

[66] A. Lawrence , “Evaluating the Effectiveness of Public Health Measures During Infectious Disease Outbreaks: A Systematic Review,” Cureus 16, no. 3 (2024): e55893. 38595888 10.7759/cureus.55893 PMC11003486

[67] M. J. Azevedo and M. J. Azevedo , “The State of Health System(s) in Africa: Challenges and Opportunities,” Historical Perspectives on the State of Health and Health Systems in Africa, the Modern Era 2 (2017): 1–73.

[68] O. J. Okesanya , G. Eshun , B. M. Ukoaka , et al., “Water, Sanitation, and Hygiene (WASH) Practices in Africa: Exploring the Effects on Public Health and Sustainable Development Plans,” Tropical Medicine and Health 52, no. 1 (2024): 68. 39385262 10.1186/s41182-024-00614-3 PMC11463047

