# Supplementary material for: The Role of Mathematical Modelling in Predicting and Controlling Infectious Disease Outbreaks in Underserved Settings: A Systematic Review and Meta‐Analysis
Source: Public Health Chall. 2025 Sep 13;4(3):e70116. doi: 10.1002/puh2.70116 (PMC12433244; doi:10.1002/puh2.70116)
Supplement: Supplementary file 1 — Search strategy [file PUH2-4-e70116-s004.docx]

**Supplementary file 1**

**Search strategy:** "models, theoretical"[MeSH Terms] OR ("models"[All Fields] AND "theoretical"[All Fields]) OR "theoretical models"[All Fields] OR ("mathematical"[All Fields] AND "models"[All Fields]) OR "mathematical models"[All Fields] OR (("determinist"[All Fields] OR "deterministic"[All Fields] OR "deterministically"[All Fields] AND ("model"[All Fields] OR "model s"[All Fields] OR "modeled"[All Fields] OR "modeler"[All Fields] OR "modeler s"[All Fields] OR "modelers"[All Fields] OR "modeling"[All Fields] OR "modelings"[All Fields] OR "modelization"[All Fields] OR "modelizations"[All Fields] OR "modelize"[All Fields] OR "modelized"[All Fields] OR "modelled"[All Fields] OR "modeller"[All Fields] OR "modellers"[All Fields] OR "modelling"[All Fields] OR "modellings"[All Fields] OR "models"[All Fields])) OR ("stoch model"[Journal] OR ("stochastic"[All Fields] AND "models"[All Fields]) OR "stochastic models"[All Fields]) OR ("agent-based"[All Fields] AND ("model"[All Fields] OR "model s"[All Fields] OR "modeled"[All Fields] OR "modeler"[All Fields] OR "modeler s"[All Fields] OR "modelers"[All Fields] OR "modeling"[All Fields] OR "modelings"[All Fields] OR "modelization"[All Fields] OR "modelizations"[All Fields] OR "modelize"[All Fields] OR "modelized"[All Fields] OR "modelled"[All Fields] OR "modeller"[All Fields] OR "modellers"[All Fields] OR "modelling"[All Fields] OR "modellings"[All Fields] OR "models"[All Fields]))) AND ("communicable diseases"[MeSH Terms] OR ("communicable"[All Fields] AND "diseases"[All Fields]) OR "communicable diseases"[All Fields] OR ("infectious"[All Fields] AND "diseases"[All Fields]) OR "infectious diseases"[All Fields] OR ("communicable diseases"[MeSH Terms] OR ("communicable"[All Fields] AND "diseases"[All Fields]) OR "communicable diseases"[All Fields])) AND ("poverty"[MeSH Terms] OR "poverty"[All Fields] OR ("low"[All Fields] AND "income"[All Fields]) OR "low income"[All Fields] OR ("poverty"[MeSH Terms] OR "poverty"[All Fields] OR "poverty s"[All Fields]) OR ("low socioeconomic status"[MeSH Terms] OR ("low"[All Fields] AND "socioeconomic"[All Fields] AND "status"[All Fields]) OR "low socioeconomic status"[All Fields])) Filters: in the last 10 years
