# Supplementary material for: The Role of Mathematical Modelling in Predicting and Controlling Infectious Disease Outbreaks in Underserved Settings: A Systematic Review and Meta‐Analysis
Source: Public Health Chall. 2025 Sep 13;4(3):e70116. doi: 10.1002/puh2.70116 (PMC12433244; doi:10.1002/puh2.70116)
Supplement: Supplementary file 2 — Characteristics and findings of the studies included in the review [file PUH2-4-e70116-s002.docx]

**Supplementary file 2**

Characteristics and findings of the studies included in the review

| **Author and year of publication** | **Title of the study** | **Aim of the study** | **Study design** | **Study setting** | **Disease studied** | **Type of mathematical model used** | **Study findings** | **Barriers encountered** |
| --- | --- | --- | --- | --- | --- | --- | --- | --- |
| Manore et al. 2014 | Comparing dengue and chikungunya emergence and endemic  transmission in A. aegypti and A. albopictus | To understand the differences in transient and endemic behaviour of chikungunya and dengue | Mathematical modelling | Reunion island | Chikungunya and dengue fever | Deterministic model. | This study adapted a transmission model for chikungunya and dengue to analyse their spread through *Aedes aegypti* and *Aedes albopictus* mosquitoes, highlighting key factors that impact outbreak size and timing. Early and rapid responses were shown to be effective, especially for dengue with *A. albopictus* and chikungunya with *A. aegypti*. Important control measures include reducing mosquito biting rates, vector-to-host ratios, and mosquito lifespan; however, chikungunya's newer strain may require broader, combined interventions due to its shorter incubation period. Environmental changes, like climate shifts and mosquito population dynamics, could also increase outbreak risks, underscoring the need for integrated strategies that target multiple aspects of transmission simultaneously to effectively reduce epidemic impacts | Parameter Assumptions: Some baseline values, like the vector-to-host ratio and maximum bites, may not accurately represent all regions, as these parameters can vary significantly.  Seasonality: The model does not account for seasonal changes, which can impact mosquito density and influence the persistence and peak size of outbreaks.  Multiple Strains and Co-Circulation: It does not consider scenarios where chikungunya and dengue might co-circulate, nor does it include multiple dengue serotypes, which can interact and affect immunity levels.  Population Dynamics: The model assumes closed populations for humans and mosquitoes, ignoring the role of human movement, which can introduce or reintroduce the virus into different regions.  Sylvatic Cycles: Sylvatic (wild animal) cycles, which may contribute to virus persistence in some areas, are not included in the analysis. |
| Lemanski et al. 2020 | Coordination among neighbours improves the efficacy of Zika control despite economic costs | To examine how different types of coordination costs and different surveillance methods jointly influence the effectiveness of larval control | Mathematical modelling | Latin America, Caribbean, Southeast Asia | Mosquito -borne viral infections (Zika, Dengue and chikungunya) | Deterministic model | The findings suggest that coordinating surveillance and control efforts among neighbouring areas can significantly improve outcomes in managing arboviral epidemics (such as Zika, dengue, and chikungunya), even when factoring in the costs of coordination. However, the benefits of coordination depend on two key factors: the nature of the costs involved and the sensitivity of the surveillance methods. | Resource Constraints in Surveillance: In many low-income regions, there is a shortage of funds and trained personnel for high-sensitivity surveillance, which is crucial for efficient vector control. Without highly sensitive surveillance, effective monitoring and timely control efforts are more challenging, especially for widespread mosquito-borne diseases.  Simplification of Mosquito Movement Patterns: The study assumes that mosquito movement occurs only between adjacent patches. Mosquito migration patterns may vary depending on geographic features, urbanization, and climate, potentially affecting the scale and coordination of control efforts.  Focus on Larval Control: The study primarily examines larval control due to its popularity in local and community control programs. However, adult control (adulticides) may also be effective in reducing mosquito populations. The exclusion of adult control from the study limits its generalizability, as real-world mosquito control programs often use a mix of larvicides and adulticides. |
| Verguet et al .2015 | Controlling measles using supplemental immunization activities:  A mathematical model to inform optimal policy | To inform country policymakers about the scheduling of Supplemental immunization activities (SIAs) to  achieve measles control | Mathematical modelling | India, Nigeria | Measles | Deterministic | The findings suggest that single supplemental immunization activities (SIAs) are insufficient to control measles outbreaks in high-burden countries. However, regular supplemental immunization (SIAs) at high coverage can control measles transmission, with the optimal timing of these campaigns depending on local demographics and routine vaccination coverage (MCV1).  The model highlights the importance of periodic SIAs, tailored to each country's conditions, to effectively control measles. | Lack of reliable data for measle notification due to underreporting and disparity in reporting quality  The model assumes the same vaccine effectiveness across all regions, though, effectiveness can differ based on healthcare quality, storage conditions, and population health. These differences can influence how well measles is controlled in each area  The model does not consider the correlation between the first dose of the measles-containing vaccine (MCV1) coverage and Supplementary Immunization Activity (SIA) coverage. Often, children who receive MCV1 may also be easier to reach through SIA, but this dynamic is not reflected in the model.  The model does not incorporate projected changes in birth rates or immunization rates, as these are difficult to predict accurately. Variations in these rates could impact measles transmission and the timing of future immunization efforts, potentially affecting the effectiveness of control measures. |
| Routledge et al. 2018 | Modelling the impact of larviciding on the population dynamics and biting rates of Simulium damnosum (s.l.): implications for vector control as a complementary strategy for onchocerciasis elimination in Africa | To identify how frequently and for how long vector control should be continued to sustain substantive reductions in vector biting. | Mathematical modelling | Ghana | Onchocerciasis | Deterministic model | The study's modelling findings suggest that focal vector control can be effective in blackfly (Simulium species) breeding sites if certain conditions are met. Key factors for success include:  Achieving and maintaining high larvicidal efficacy.  Regular and frequent larvicidal applications, ideally on a weekly basis for S. damnosum breeding sites.  Sustained control efforts over time.  The findings also indicated that combining vector control measures with ivermectin mass drug administration (MDA) can enhance efforts to eliminate onchocerciasis. | Lack of detailed, locale-specific field data on blackfly populations Variation in vectorial characteristics across different cytotaxa are significant limitations in effectively planning vector control.  The model currently simplifies the effect of vector control by reducing the annual biting rate of blackfly populations without capturing the more complex dynamics of larviciding, such as varying durations and frequencies of treatment. This limits the ability to model the detailed impact of vector control on blackfly population dynamics.  The model for forest areas does not account for adult fly mortality, migration, or movement between neighbouring areas, which could affect the effectiveness of vector control. This omission makes it difficult to fully assess the impact of reinvasion from surrounding untreated areas and the potential for migration-related dynamics in transmission zones. |
| Pongsumpun and Tang 2014 | Dynamics of a New Strain of the H1N1 Influenza A Virus Incorporating the Effects of Repetitive Contacts | The aim of the study is to investigate the transmission dynamics of a new strain of the H1N1 influenza A virus in humans, specifically considering the effects of repetitive social contacts | Mathematical modelling | Thailand | H1N1 | Deterministic model | Increasing the amount of repetitive contacts  leads to a decrease in the peak numbers of exposed and infectious human.  Both an increase in the virulence of the virus and a higher fraction of infectious individuals placed in quarantine lead to a decrease in the number of infectious humans at equilibrium. This suggests that more severe strains of the virus and effective quarantine measures can help control infection levels.  The study advocates for a combination of quarantine measures and public education efforts to manage infectious diseases effectively, emphasizing that policymakers should remain adaptable in their strategies as the virus evolves. | Reliance on Early Data: The predictions for transmission and death rates are based on data collected at the start of outbreaks. Early data are often limited or inaccurate, so these estimates may not be very reliable  Simplified Assumptions: The model uses fixed rates for contact, quarantine, and mortality. In reality these rates can vary and depend on many factors, so this simplification may not fully capture the true spread of the disease. |
| Reddy et al. 2021 | Cost-effectiveness of public health strategies for COVID-19 epidemic control in South Africa: a microsimulation  modelling study | 1.To compare the medical outcomes and costs of various COVID-19 control measures  2. To inform policy decision-making by projecting clinical outcomes, cost-effectiveness, and budget impact of alternative control strategies, focusing on interventions either proposed or currently in use in South Africa. | Mathematical modelling | South Africa, KwaZulu Natal | Covid-19 | Stochastic modelling | In South Africa, public health strategies that include household contact tracing, isolation, mass symptom screening, and quarantining household contacts who test negative for COVID-19 would significantly lower the number of deaths caused by the disease. Additionally, these strategies would offer good value for the resources spent, therefore the benefits outweigh  the costs.  Policymakers should adopt combined approach for controlling the COVID-19 epidemic, which includes health-care testing, contact tracing, isolating confirmed cases, conducting mass symptom screening, and quarantining household contacts of infected individuals to maximize efficiency. | Assumptions on Age and Comorbidities: The model assumes that age and non-communicable disease prevalence in South Africa are similar to patterns in the USA, which may not fully capture the influence of local factors like HIV prevalence on COVID-19 severity.  Limited Data on HIV Impact: Due to a lack of sufficient data, the model assumes no increased severity of COVID-19 due to HIV, which may underestimate COVID-19 risks in HIV-endemic regions.  Localized Cost Estimates: The costs for intervention strategies were based on local vendors in KwaZulu-Natal, which may not apply to other regions or reflect full-scale implementation expenses.  Assumptions on Intervention Efficacy: The model uses hypothetical estimates for some intervention outcomes based on limited data and expert opinion, which may not be accurate for all contexts.  No Economic Sector Analysis: Broader economic impacts beyond healthcare, such as on other economic sectors, were not included in the analysis, limiting insights into the overall societal costs of interventions.  Assumption of Random Contact Patterns: The model assumes that people mix randomly, which does not reflect real-life situations where contact patterns vary within communities. |
| Denes and Gumel 2019 | Modelling the impact of quarantine during an outbreak of Ebola virus disease | To design a new model for realistically assessing the population-level impact of quarantine on the spread and control of the 2014-2015 EVD outbreaks in the three affected Western African countries. | Mathematical modelling | Guinea, Liberia, Sierra Leone | Ebola | Deterministic | The study identifies key factors that influence the control of Ebola Virus Disease (EVD), including the community contact rate, transmission probability per contact, and the time before burying Ebola-deceased individuals, all of which significantly impact the disease burden. Reducing factors such as minimizing contact with suspected cases and shortening burial delays can help curb the spread of the virus. While quarantining susceptible individuals has a smaller effect on the overall infection rate, minimizing contact during quarantine is essential to lowering the reproduction number and achieving effective disease control.  The findings suggest that quarantine can still play an important role in controlling EVD if it is implemented with high coverage and effectiveness, especially when used alongside other control measures. | The model assumes quarantine is not fully effective, meaning disease transmission can still occur during quarantine, limiting the effectiveness of quarantine measures unless they are implemented with high efficacy.  Backward Bifurcation: The introduction of imperfect quarantine leads to a backward bifurcation, which complicates disease control. This phenomenon implies that reducing the basic reproduction number (R₀) to below one may not be sufficient to eliminate the disease; it requires additional efforts to lower it further outside the backward bifurcation region.  Limited Impact of Quarantine for Susceptible Individuals: The sensitivity analysis indicates that quarantining susceptible individuals has a relatively small effect on reducing the number of infected cases, which may limit the overall impact of quarantine measures focused on susceptible individuals.  Reliance on High Coverage and Efficacy: For quarantine to be effective, it must have high coverage and efficacy in minimizing disease transmission during the quarantine period. Without these high standards, quarantine measures alone may be insufficient for controlling or eliminating the disease.  Influence of Community Contact Rates and Burial Delays: The community contact rate, transmission probability per contact, and mean time before burial are influential factors for disease transmission. Quarantine alone may not fully address these external factors, and additional control measures may be necessary |
| Azeez et al. 2016 | Seasonality and Trend Forecasting of Tuberculosis Prevalence Data in Eastern Cape, South Africa, Using a Hybrid Model | To compare a hybrid model to forecast the TB incidence epidemic with an existing model and to assess the model seasonality trends in South Africa | Modelling | South Africa, Eastern Cape province | Tuberculosis | Stochastic model | The study's findings suggest that there is unlikely to be significant improvement in reducing the high TB incidence in the Eastern Cape in the near future. The projected outcomes indicate a slight rise in reported TB cases in the coming years. These results highlight the need for intensified efforts and more effective interventions to control TB in the region urgently. | The models were based on data from 2010 to 2015 and verified with only one additional year of TB prevalence, meaning the results should be interpreted cautiously and revisited with more extensive time series data.  Missing Data on Key Factors: Important factors like climate, migration, and demographic data, which might influence TB spread, weren’t included in the model due to lack of available data. This means the model may not fully capture all the reasons behind TB progression. |
| Levy et al.2017 | Modelling the role of public health education in Ebola virus disease outbreaks in Sudan. | To assess how behaviour change induced by education can alter the dynamics of an outbreak of EVD | Mathematical modelling | Sudan | Ebola | Deterministic model | Educated vs. Uneducated Populations: Populations with higher levels of education and awareness about EVD exhibit significantly lower transmission rates. In simulations, educated individuals who take proper precautions have a lower chance of spreading the disease.  Cultural Practices: The study notes that cultural practices, such as handling deceased individuals, contribute to the spread of EVD, and breaking these practices through education helps control outbreaks.  Health Care Preparedness:  The preparedness of healthcare systems and the availability of trained professionals also play a key role in mitigating the outbreak.  Well-equipped facilities and trust in healthcare systems can reduce the disease burden. | Assumptions About Education: The study assumes that people who are more educated about Ebola are less likely to get infected, but this could be an oversimplification. Different factors, like access to resources, also play a role in reducing infections  Reliability of Old Data: The research relies on outbreak data from 1976 and 1979 in Sudan. Since these are older outbreaks, some details may not be as precise as those from more recent cases.  Cultural Factors: The model does mention cultural practices that spread Ebola but doesn’t fully consider how hard it is to change these traditions in different regions.  Healthcare System Differences: The study highlights the importance of prepared healthcare systems but doesn’t explore specific factors, like training or resources, that vary between regions and can affect outbreak response.  Sensitivity to Data Changes: Small changes in data, like infection or recovery rates, can lead to big differences in the model's predictions, which might limit its reliability.  Generalizing Education Levels: By grouping people into “educated” and “uneducated,” the study may miss important differences within these groups, as some people may know more about Ebola than others. |
| Nagpal et al. 2024 | Seasonal variations in social contact patterns in a rural population in north India: Implications for pandemic control. | To analyse seasonal variations in social contact patterns by age, gender, and employment in rural north India to enhance COVID-19 transmission modelling and prediction accuracy. | Mathematical modelling | India (Rural Ballabgarh) | Covid-19 | Agent-based model | The study found that social contact patterns in rural north India change significantly with the seasons, with people having the most and longest interactions in winter, followed by summer and monsoon.  Children and teenagers aged 0–19 had the highest number of social contacts and played a central role in community interactions.  Most physical contacts took place in high-contact settings, like schools and public transport, where over 80% of interactions involved physical touch.  Age and gender also shaped contact patterns, especially among younger people. Additionally, factors such as season, age, job status, and weekday influenced the amount and length of contact. These seasonal patterns were used to create a model to better predict COVID-19 spread in the area. | The use of contact diaries may lead to recall bias, inaccuracies in reporting group sizes, and overestimations of time spent on certain activities.  Short-duration contacts (<5 minutes) are often forgotten, and reporting fatigue could affect accuracy due to the longitudinal survey design.  Sampling from a convenience sample with a high density of middle-aged and elderly individuals may limit the generalizability to other regions.  Data was collected before COVID-19, which may impact its relevance to current social contact patterns.  Lastly, the findings reflect seasonal contact changes within a single year rather than longer-term trends. |
| Hamley et al. 2021 | What does the COVID-19 pandemic mean for the next decade of onchocerciasis control and elimination? | To predict how missed or delayed mass drug administration will impact short-term epidemiological trends and the prospects for onchocerciasis elimination by 2030. | Mathematical modelling | Liberia, Guinea, Sierra Leone, Mali, Senegal, Nigeria | Onchocerciasis | Stochastic models | Vulnerability of Shorter Treatment Histories: Both the EPIONCHO-IBM and ONCHOSIM models indicated that MDA programs with shorter treatment histories are most vulnerable to interruptions caused by the COVID-19 pandemic. This vulnerability is particularly pronounced if MDA cannot safely resume in 2021. This could lead to a resurgence of local infection, especially in high-endemicity settings, reducing the likelihood of achieving the elimination of transmission by 2030.  Programs with longer histories of annual MDA, which have achieved and maintained 65% coverage, are predicted to be less affected by these interruptions. However, they are still vulnerable, particularly if the initial level of endemicity was high, which indicates more favourable transmission conditions.  The study suggests that young children in highly endemic areas may face increased levels of infection due to missed MDA rounds, especially with a 2-year interruption. | Uncertainty in Child Infection Levels: The models differ in predicting how many young children may be infected, especially those under 5 years old, which affects overall predictions on disease control.  Simplified View of Treatment History: The study assumes past treatment levels strongly influence how current disruptions affect infection rates, but this may oversimplify real-world outcomes. |
| Adiga et al. 2017 | Disparities in spread and control of influenza in slums of Delhi: findings from an agent-based modelling study | To study the role of slums in the spread and control of infectious diseases | Mathematical modelling | Slums of Delhi, India | Influenza | Agent-based model | The study found that when slum-specific factors are ignored, the effectiveness of vaccination in reducing infections and controlling an epidemic can be overestimated by 30% to 55%.  Slum populations experience higher infection rates compared to non-slum areas, regardless of the intervention used. However, vaccination is more effective in slums than social distancing strategies.  This highlights the need to account for slum conditions when planning interventions to control disease spread | The study assumes that people in both slum and non-slum areas have similar immunity levels, even though those in slums, due to larger family sizes and greater microbial exposure, may have higher immunity to respiratory infections. This oversight might affect the accuracy of the model’s predictions. |
| Njankou and Nyabadza 2022 | Modelling the potential influence of human migration and two strains on Ebola virus disease dynamics. | To develop a mathematical model to assess the potential introduction, spread, and interaction of new Ebola virus strains within a country, considering factors like infected migration, mutation, and cross-border movement control. | Mathematical modelling | Guinea, Liberia and Sierra Leone | Ebola | Deterministic | This study explored how different patterns of infected individuals entering a country affect the spread of two Ebola strains. When infected people arrive continuously, both strains can spread quickly, making control measures harder to implement. However, if infected individuals enter occasionally or in bursts, it's easier to manage outbreaks because there’s more time to respond, and fewer cases of the new strain emerge. Additionally, if a new strain arises locally due to mutation, the more infectious strain will dominate. Overall, the findings suggest that controlling Ebola is more feasible when infected individuals arrive in bursts rather than continuously, as this allows more effective intervention and can prevent higher death tolls in multi-strain outbreaks. | Limited Scope of Strain Characteristics: The model assumes that the new strain (EVD2) is less infectious than the resident strain (EVD1). However, if the new strain behaves differently (e.g., it could mutate to become more infectious), the model’s predictions might not hold true.  Focus on Strain Competition, Not Other Factors: The study focuses mainly on strain competition and immigration patterns, but other factors like public health infrastructure, healthcare responses, and population behavior are not fully integrated into the model.  Simulation Limits: The results are based on numerical simulations that may not fully capture the variability of real-world situations, such as different outbreak intensities, varying immunity levels in the population, and different healthcare system responses.  Simplified Control Measures: The study suggests control measures but assumes that they can be implemented effectively within the timeframes provided by the model. In reality, factors like infrastructure, resource availability, and political or social challenges could delay or limit the effectiveness of control measures. |
| Fauzi et al. 2024 | Assessing the impact of booster vaccination on diphtheria transmission: Mathematical modelling and risk zone mapping | To evaluate how effective booster vaccinations are in reducing the spread of diphtheria using mathematical modelling  To analyse the spatial distribution of diphtheria cases in West Java, identifying areas where the infection is most common. | Mathematical modelling | West Java, Indonesia | Diphtheria | Deterministic model | The findings indicated that disruptions in vaccination efforts during the COVID-19 pandemic may have contributed to a post-pandemic rise in diphtheria cases.  The model’s findings indicated a basic reproduction number above 1, pointing to a heightened outbreak risk that could be controlled through improved booster vaccination coverage.  The spatial analysis pinpoints high-risk zones in the western, central, and southern parts of West Java, areas marked by both high-density populations and rural regions with sparse populations. The findings underscore the importance of targeted efforts by local governments and health authorities in these regions to prevent major outbreaks and enhance public health interventions. | lack of complete data, especially regarding the vaccination status of infected individuals, which is important for evaluating how well vaccinations prevent diphtheria. There may also be unreported diphtheria cases due to issues with data recording.  The mathematical model used is quite basic, relying solely on the SIR framework. To improve the analysis, more complex models could be developed that include additional factors related to diphtheria transmission, such as different levels of vaccination coverage. |
| Kuddus et al. 2024 | Cost‑effectiveness analysis of COVID‑19 intervention policies using a mathematical model: an optimal control approach | To develop a COVID-19 model to understand the transmission dynamics of COVID-19 in Bangladesh | Mathematical modelling | Bangladesh | Covid-19 | Deterministic model | The findings suggest that a three-pronged approach combining transmission control, treatment, and vaccination is the most cost-effective option compared to single or double intervention methods and may effectively decrease overall infections. Additionally, other policies can be considered for managing COVID-19 based on available funding and the decisions of policymakers | Underreporting of Cases: Bangladesh's COVID-19 surveillance system might miss some cases, resulting in biased estimates due to this underreporting.  Data Collection Methods: The study indicates that the existing data collection methods may not be adequate, potentially compromising the accuracy of the information related to COVID-19. |
| Pearson et al. 2021 | COVID-19 vaccination in Sindh Province, Pakistan: A modelling study of health impact and cost-effectiveness | To assess the health impact, economic impact, and cost-effectiveness of COVID-19 vaccination in Sindh Province, Pakistan, using a combined epidemiological and economic model. | Modelling study | Sindh province, Parkistan | COVID-19 | Deterministic model | The model predicts that a COVID-19 vaccine rollout in Sindh, Pakistan, could prevent large numbers of infections and deaths, making it potentially cost-effective. In a typical scenario, if 20% of the population is vaccinated within a year with a vaccine that’s 70% effective for about 2.5 years, it could prevent around 900,000 cases and 10,000 deaths. This approach would cost an additional $2 million compared to not vaccinating, and it would save 70,000 years of healthy life at a cost of $28 per year saved, assuming each vaccine dose costs $1. If the program is extended to 5 or 10 years, it could save more lives but at higher costs per year of healthy life saved.  The model found that targeting only older adults (65+) was as cost-effective as vaccinating everyone aged 15 and older, although the broader approach would prevent more cases across all ages. This analysis is more relevant for low- and middle-income countries, which have similar population age structures, economic limitations, and high COVID-19 spread in the past, compared to high-income countries.  The study also notes that if vaccines become more expensive or less effective, their cost-effectiveness would drop. Resource limitations, such as healthcare staff and infrastructure, mean that vaccination strategies must carefully balance both short-term needs and the long-term stability of the healthcare system.  The authors recommend that vaccination planning in low-resource settings should consider both the direct benefits of vaccination and broader social and economic impacts. | Cost Concerns: The model shows cost-effectiveness if vaccine doses are affordable (e.g., $1 per dose). But if costs go up or effectiveness drops, vaccination might be much less cost-effective.  Immunity Uncertainty: The model assumes immunity from vaccines or previous infections fades, but it doesn’t fully consider new variants that might weaken vaccine effects, meaning more vaccinations could be needed.  Immunity Uncertainty: The model assumes immunity from vaccines or previous infections fades, but it doesn’t fully consider new variants that might weaken vaccine effects, meaning more vaccinations could be needed.  Broad Age Targeting: The model considers large age groups but doesn’t go into detail on targeting high-risk subgroups, which may limit understanding of the best groups to prioritize. |
| Rock et al. 2024 | The Hidden Hand of Asymptomatic Infection Hinders Control of Neglected Tropical Diseases: A Modelling analysis | To analyse the dynamics of visceral leishmaniasis (VL), gambiense human African trypanosomiasis (gHAT), and Chagas disease in the Indian subcontinent and assessing the role of asymptomatic individuals in disease transmission and evaluating the potential impact of various interventions, such as active screening, vector control, and reduced time to detection, on disease control and elimination. | Mathematical modelling | India | Visceral leishmaniasis (VL), gambiense sleeping sickness(gHAT), and Chagas disease | Deterministic model | The study found that gHAT (Gambian Human African Trypanosomiasis) could be the easiest of the three diseases (gHAT, Chagas disease, and visceral leishmaniasis) to control with a combination of existing methods, assuming an effective healthcare system in endemic areas. For Chagas disease, early treatment of asymptomatic individuals is essential to prevent disease progression and reduce community transmission, but this requires better diagnostics. In the case of visceral leishmaniasis (VL), the lack of data on the infectiousness of different stages and limited treatment options for asymptomatic cases make it more challenging to control. Overall, the study emphasized the need to target asymptomatic infections more effectively, as these individuals play a significant role in spreading disease. This approach could reduce transmission, relieve strain on health systems, and help eliminate these neglected tropical diseases if public health strategies adapt to focus on asymptomatic carriers. | There was lack of experimental or observational data to support the assumptions used in creating models and making policies about these diseases. This lack of information makes it hard to accurately predict how the diseases spread, how they behave, and how effective different interventions will be.  Broad Scope of the Model: While the model provides a general framework for vector-borne NTDs, it lacks specificity for individual diseases. It does not fully capture unique aspects, such as animal transmission in Chagas and gHAT or maternal transmission in gHAT, limiting its applicability to other complex disease transmission cycles.  Limited Understanding of Infection Dynamics: The study acknowledges that infection dynamics in vector-borne diseases are not at equilibrium. This constant shift in transmission dynamics makes it challenging to predict long-term impacts of interventions, particularly for diseases with complex cycles, like Chagas disease and visceral leishmaniasis (VL), which involve various reservoirs and co-infections.  Funding and Conflict of Interest: While funding sources and conflicts of interest are disclosed, reliance on a limited number of funders could potentially influence research directions, especially for diseases in endemic areas with underfunded health systems. |
| Lewnard et al. 2014 | Dynamics and control of Ebola virus transmission in  Montserrado, Liberia: a mathematical modelling analysis | To assess the effectiveness of expanding EVD treatment centres, increasing case ascertainment, and allocating protective kits for controlling the outbreak in Montserrado | Mathematical modelling | Montserrado, Liberia | Ebola | Deterministic model | To control the Ebola outbreak in Montserrado, far more treatment canters are needed than have been promised by international aid. Having more treatment centres would greatly lower the number of cases and deaths.  The effectiveness of these canters increases significantly if paired with fast and efficient case identification, which involves quickly finding and isolating infected individuals through contact tracing or when they seek care.  Protective kits are also helpful, especially for households that cannot access treatment centres, though their effectiveness varies from 10% to 50%. Starting these measures even two weeks sooner would have greatly reduced the spread and saved lives. Any delay in implementing these actions will allow the outbreak to worsen, making it harder to contain. | Under reporting: Ebola cases are not detected until after people start showing symptoms. Because of this delay, there is likely an undercount of actual cases, as some infected individuals might not be identified or reported in a timely manner. As a result, the true scale of the outbreak could be larger than the reported numbers suggest, which could hinder effective planning and response efforts.  Limited Community Data: The model uses general assumptions about how people mix and interact, which might not capture local variations in behaviors or health systems, especially in high-transmission areas like Montserrado.  Simplified Transmission Assumptions: The model assumes all infected individuals transmit the virus equally, although viral loads can vary between patients and at different stages of infection. This could affect the accuracy of predicted spread and intervention impact.  No Consideration of Burials: The model does not account for potential transmission during burials. In the absence of data on the infection risk from these events, the impact of interventions on this transmission route remains uncertain. |
| Baik. et al. 2022 | Symptom-based vs asymptomatic testing for controlling SARS-CoV-2 transmission in low- and middle-income countries: A modelling analysis | The aim of the study was to examine the potential epidemiological impact of different strategies for deploying lateral flow assays (LFAs) as a diagnostic tool for COVID-19 in a resource-limited setting, specifically a large Indian city. | Mathematical modelling | India | Covid-19 | Deterministic model | The study found that using 25 million lateral flow assays (LFAs) over 600 days could lower the number of COVID-19 cases by 0.44% if used for community screening and by 13% if used for testing people with symptoms in outpatient settings. The analysis showed that outpatient testing works best if at least 5% of symptomatic patients seek care and at least 10% of infections cause symptoms. However, both approaches resulted in 2% of the population being isolated unnecessarily. The researchers concluded that using LFAs for symptomatic individuals in outpatient settings would be the most effective way to reduce virus spread. To avoid unnecessary isolations, LFAs should be used for initial screening, followed by confirmatory tests. Future studies should investigate targeted community-based testing strategies, like contact tracing. | Lack of Detailed Age Data: The model didn't consider more complex vaccination strategies that target specific age groups. This is because there wasn't enough data on COVID-19 cases by age, making it hard to plan.  The model assumed that immunity from infection or vaccines lasts for a set time. It didn't fully account for uncertainties about how long people stay protected or how new virus variants could change that. |
| Zandvoort et al. 2020 | Response strategies for COVID-19 epidemics in African settings: a mathematical modelling study | The aim was to evaluate strategies for reducing COVID-19 impacts in African countries by addressing unique demographic and socio-economic factors to help balance saving lives, safeguarding healthcare, and supporting livelihoods. | Mathematical modelling | Mauritius, Nigeria, Niger | Covid-19 | Deterministic model | The study predicts that if COVID-19 epidemics are not controlled, African countries could see high infection rates, with up to 42% of people affected in the first year. Self-isolation of symptomatic individuals and physical distancing can help reduce severe cases, but their impact varies depending on the level of contact reduction and virus transmission rates. Shielding high-risk individuals, especially if they have limited contact with others, can significantly lower hospital demand and deaths. Combining self-isolation, moderate distancing, and shielding could cut mortality in half. Temporary lockdowns, though difficult to sustain, can delay the epidemic’s peak and give countries time to strengthen their healthcare response. | The model may not fully capture COVID-19's spread in African settings, where urban centres might see rapid case increases, followed by a slower rise in rural areas. This variation could affect the model’s accuracy for predicting how long the epidemic may last in these countries.  The model used estimates from Chinese data on severe cases by age but adjusted them to reflect possible differences in Africa, where younger people may be more vulnerable due to co-existing conditions like tuberculosis, HIV, and undernutrition. This could shift severe cases to younger age groups.  The model does not account for factors like population density, mobility, and healthcare capacity, which may affect transmission and the success of interventions. For example, many African countries have limited healthcare services, potentially leading to higher fatality rates. |
| Drolet et al. 2021 | Optimal human papillomavirus vaccination strategies to prevent cervical cancer in low-income and middle-income countries in the context of limited resources: a mathematical modelling analysis | To evaluate different HPV vaccination strategies to determine the most effective and efficient ways to use limited vaccine supplies in low- and middle-income countries (LMICs). | Mathematical modelling | India, Vietnam, Uganda and Nigeria | Cervical cancer | Deterministic model | The model predicted that Vaccinating girls against HPV and ensuring high vaccination rates can help eliminate cervical cancer in countries where the cancer rates are low to moderate, such as Vietnam (9 cases per 100,000) and Nigeria (34 cases per 100,000). However, in countries like Uganda, where the cancer rates are higher (57 cases per 100,000), just vaccinating may not be enough. In these places, regular cervical screening will also be needed to effectively reduce cervical cancer cases and work towards elimination. | Did not model all potential age combinations and coverage, making results general principles rather than definitive solutions.  The impact of HIV on HPV acquisition and disease progression was not included, which may affect the estimated benefits of vaccination in high-HIV-prevalence settings.  Single-Dose Efficacy Uncertainty: Although extended intervals between doses could save costs, it is uncertain if one dose will provide lasting protection. Clinical trial results on single-dose efficacy are not yet available, so the model’s predictions may change based on future findings. |
| Ladib et al.2024 | Mathematical modelling of contact tracing and stability analysis to inform its impact on disease outbreaks; an application to COVID-19 | To investigate the effect of contact tracing on containing epidemic outbreaks and slowing down the spread of transmissible diseases. | Mathematical modelling | South Korea, Brazil and Venezuela | Covid-19 | Deterministic model | The study confirmed that effective contact tracing can help control the transmission of COVID-19.  The findings suggested that improving the effectiveness of contact tracing requires either increasing the detection of initial cases or enhancing the tracking of infected individuals. However, investing excessively in non-tracing detection methods may not be beneficial beyond a certain point, highlighting the importance of optimal resource management.  The study found that changes in the reproduction number are linearly linked to the effectiveness of contact tracing which implies that reproduction number depends proportionally on the performance of contact tracing relative to non-tracing detection.  The model indicates that for contact tracing to remain effective, an optimal approach may be necessary, balancing between detecting new cases and managing resources, such as the workforce size dedicated to tracing. | Incomplete Proof for Reproduction Number: While  𝑅𝑒 ​was calculated and showed promising results, a rigorous proof for its use as the effective reproduction number was not established, meaning the findings need further validation.  Simplified Detection Interactions: The model does not fully incorporate varying detection probabilities across different disease stages, which could affect how accurately it represents the real-world effectiveness of contact tracing.  Limitations of Parameter Fitting: The model was applied to only a few countries and over a specific period, which may limit its broader applicability across varying demographics and stages of an outbreak. |
| Mahmud 2021 | Megacities as drivers of national outbreaks:  The 2017 chikungunya outbreak in Dhaka, | To determine the role of human mobility on the spatial spread of chikungunya | Mathematical modelling | Dhaka, Bangladesh | Chikungunya | Deterministic model | The results indicated the impact of large-scale population movements, particularly during holidays, on the spread of infectious diseases, and emphasized the value of real-time data from mobile phones for outbreak analysis and forecasting.  The study revealed that the incidence of chikungunya was much higher than official reports indicated, driven by high population density and mobility within the city. These factors contributed to spatial variations in incidence, with densely populated areas experiencing more cases.  The outbreak peaked during the Eid holidays, highlighting how large-scale population movements can facilitate the spread of infections beyond urban centers.  A mechanistic transmission model estimated a human-to-human reproduction number of 4.20 and indicated that about 22% of infections were asymptomatic. | Chikungunya incidence was based on survey data rather than population-based surveillance, which may not be representative of the entire population.  Self-reported symptoms and a lack of lab confirmation for all suspected cases could bias the estimates.  There may be bias in the mobility data because it was based on mobile phone users, who may not fully represent the general population. Nonetheless, the large portion of the city’s population covered by mobile subscribers means the data likely captures a significant trend, especially during the Eid holiday travel spike.  The study couldn’t validate its estimates for cases coming into Dhaka because there is limited surveillance data outside the city. Without enough data from other areas, it wasn’t possible to build a detailed model of population movement and disease spread. Despite this, the results highlight how real travel patterns differ from simpler models and show the importance of using mobility data to improve understanding of disease spread across Bangladesh. |
| Fung 2024 | Long-term effects of non-pharmaceutical interventions on total disease  burden in parsimonious epidemiological models | To help resolve the uncertainty by providing quantitative estimates of the impact of NPIs on the long-term total disease burden for non-target diseases, to aid in assessing the impact and formulation of policies in  public health. | Mathematical modelling | Gambia, Florida and Singapore | Respiratory diseases | Deterministic model | This study suggests that non-pharmaceutical interventions (NPIs) are likely to reduce the long-term disease burden of non-target seasonal respiratory diseases.  The study suggested that to manage the risk of large outbreaks following the removal of non-pharmaceutical interventions (NPIs), health policies should focus on significantly increasing vaccination coverage | Limitations of Simplified Model: The basic model assumed a fixed rate of immunity loss, while immunity in reality decreases gradually over time.  Simplified Models: The models used are basic versions that don’t include many real-world details. This means that they might miss important parts of how diseases actually spread, making the results less reliable in real settings.  Ignoring Multiple Strains: These models only consider one type of a disease. But in real life, a virus can have multiple strains or types.  No Consideration of Geography: The models don’t take location into account. But where people are and how they move affects how diseases spread. A model that includes geography (like mapping how people interact in certain areas) would help show how diseases spread across different places. |
| Liu 2023 | Assessing the impacts of COVID-19 vaccination program's timing and speed on health benefits, cost-effectiveness, and relative affordability in 27 African countries | To assess the impact of vaccination program timing husing an epidemiological and economic  model. | Mathematical modelling | Africa | Covid-19 | Deterministic model | The study found that starting COVID-19 vaccination programs early brought the greatest health benefits and was the most cost-effective. Although a fast vaccine roll-out also improved health outcomes, it wasn’t always the most cost-effective, especially when vaccinating many less vulnerable people. The most significant health impact was seen in vaccinating older adults. Cost-effectiveness was also influenced by factors like a country's income level, the proportion of people over 60, and the number of individuals already less at risk when vaccinations began. Overall, vaccination programs with lower costs relative to the country’s economic capacity were generally more affordable. | The study may have underestimated vaccine delivery costs, as social outreach efforts could increase costs, and actual data on the costs of different vaccine programs was unavailable.  The model assumes that the only reason for low vaccine uptake is limited supply, but it doesn’t consider other factors like vaccine hesitancy, which could also slow down uptake. |
| Das 2024 | Exploring the dynamics of monkeypox transmission with data-driven methods and a deterministic model | To explore the dynamics of monkeypox transmission | Mathematical modelling | Brazil, USA | Monkeypox | Deterministic model | Higher vaccination rates among at-risk groups significantly reduce Mpox transmission rates, helping to flatten infection curves and lower the basic reproduction number.  Reducing close contact in high-risk groups was shown to be an effective measure for controlling the spread of Mpox.  Projections indicated that Mpox cases were likely in a decline phase as of July 2023, with similar trends observed in regions like Brazil. | Data quality and completeness |
| Shi et al 2020 | Inference and prediction of malaria  transmission dynamics using time series data | To estimate and predict the dynamics of malaria transmission based on time series prevalence data using data-driven stochastic model | Mathematical modelling | China, Tengchong and Longling, Yun-nan province, | Malaria | Stochastic model | the model provided reasonable estimates of transmission dynamics for P. vivax in the study areas.  It successfully captured the effects of meteorological changes and seasonal variations in imported cases on malaria transmission.  The one-step-ahead forecasting method demonstrated good performance, showing the model's potential to predict future infections. | Parameter Limitations: Some key numbers used in the model, like mosquito density, were pulled from other studies rather than being specific to the local area studied.  Seasonal Assumptions on Imported Cases: The model assumes a seasonal pattern for malaria cases brought in from outside, which might not be accurate and affects the model's precision. Understanding where and when people bring in cases could improve the model’s predictions.  Simplified Disease Processes: The model is based on an older malaria model and misses some important factors, like relapses of Plasmodium vivax malaria and incubation delays. Adding these would make the model more complete. |
