# Supplementary figures and images for: The Role of Mathematical Modelling in Predicting and Controlling Infectious Disease Outbreaks in Underserved Settings: A Systematic Review and Meta‐Analysis
Source: Public Health Chall. 2025 Sep 13;4(3):e70116. doi: 10.1002/puh2.70116 (PMC12433244; doi:10.1002/puh2.70116)

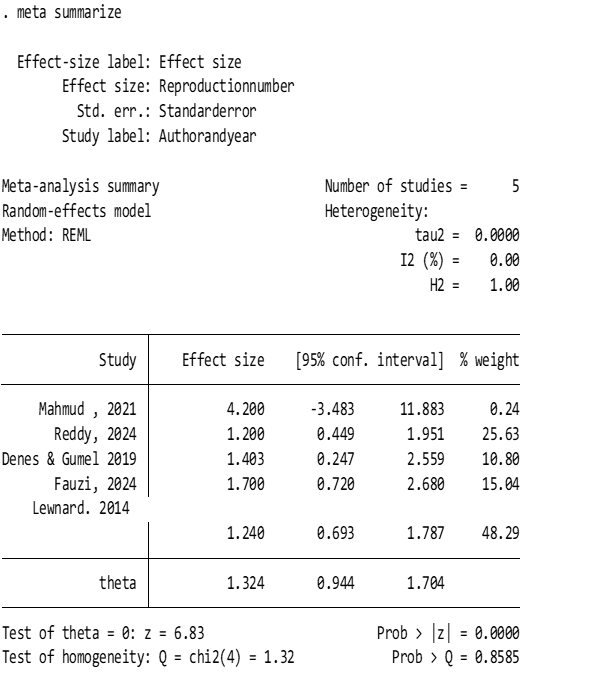

Supplement: Supplementary file 3 — Summary of meta‐analysis results [file PUH2-4-e70116-s005.tif]

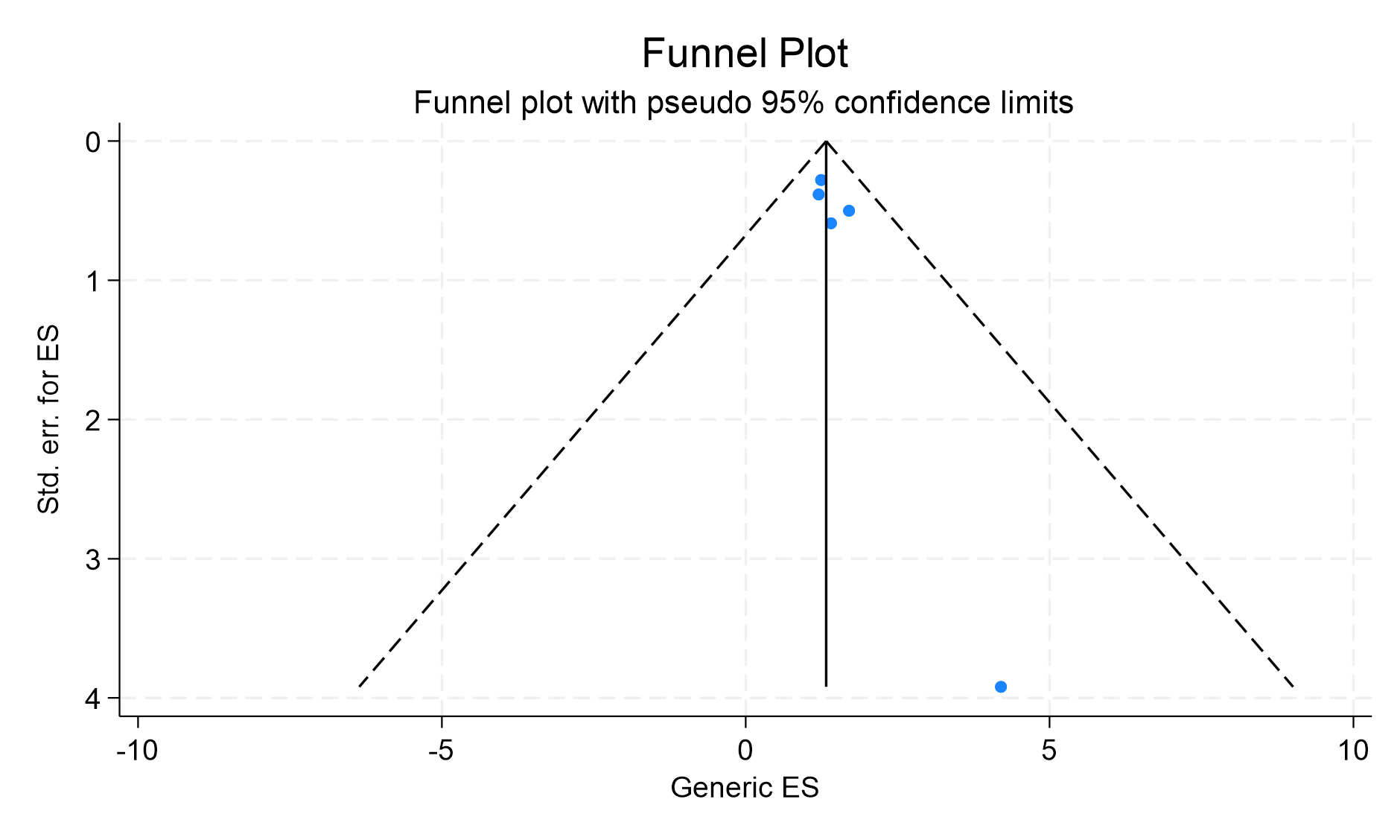

Supplement: Supplementary file 4 — Funnel plot representing publication bias assessment [file PUH2-4-e70116-s001.tif]

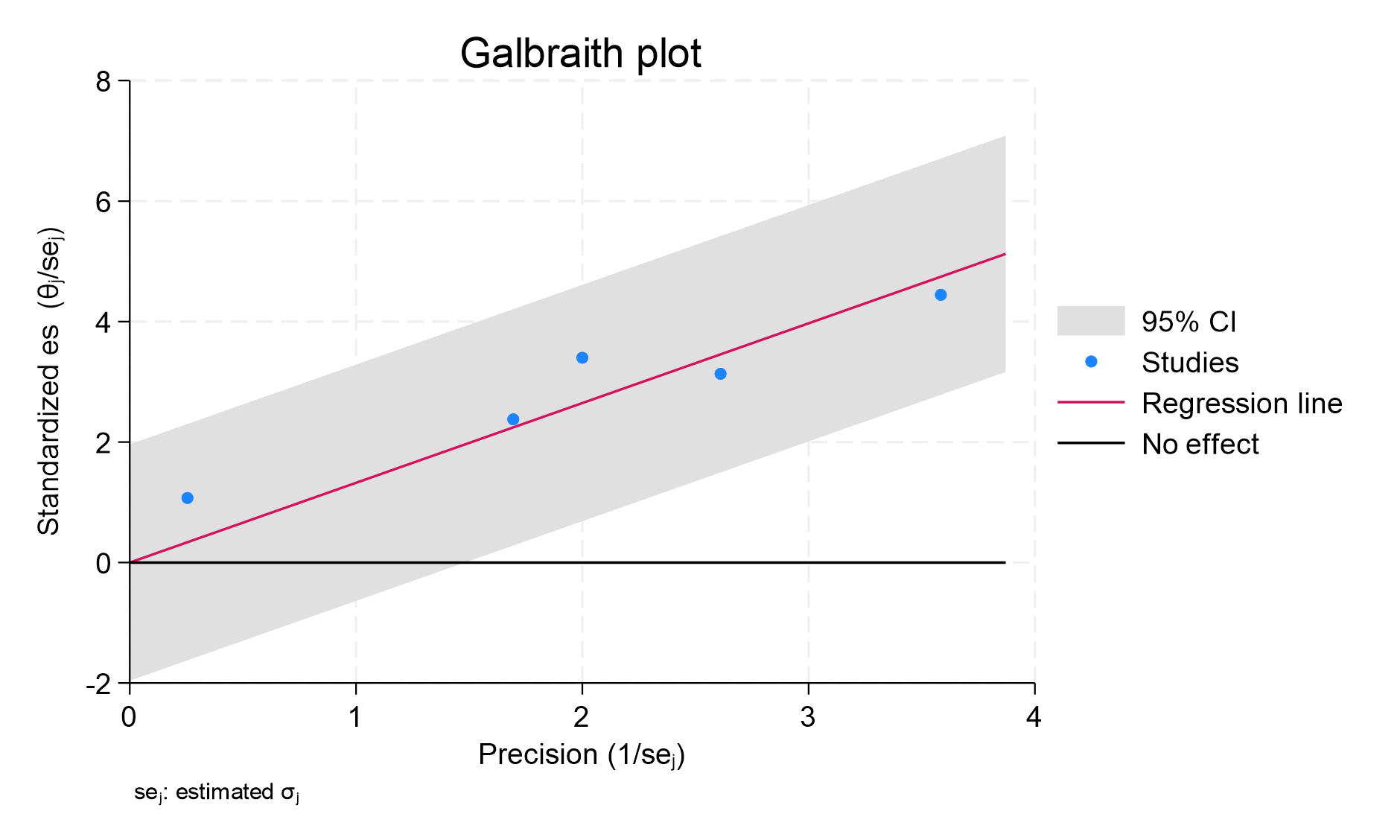

Supplement: Supplementary file 5 — Galbraith plot to identify outliers [file PUH2-4-e70116-s003.tif]
